# Supplementary material for: LIN28B Impairs the Transition of hESC-Derived β Cells from the Juvenile to Adult State
Source: Stem Cell Reports. 2019 Dec 26;14(1):9–20. doi: 10.1016/j.stemcr.2019.11.009 (PMC6962644; doi:10.1016/j.stemcr.2019.11.009)
Supplement: Document S1. Supplemental Experimental Procedures and Figures S1–S3 [file mmc1.pdf]

Stem Cell Reports, Volume *14*

Supplemental Information

LIN28B Impairs the Transition of hESC-Derived β Cells from the Juvenile to Adult State

Xin Zhou, Gopika G. Nair, Holger A. Russ, Cassandra D. Belair, Mei-Lan Li, Mayya Shveygert, Matthias Hebrok, and Robert Blelloch

Figure S1

A

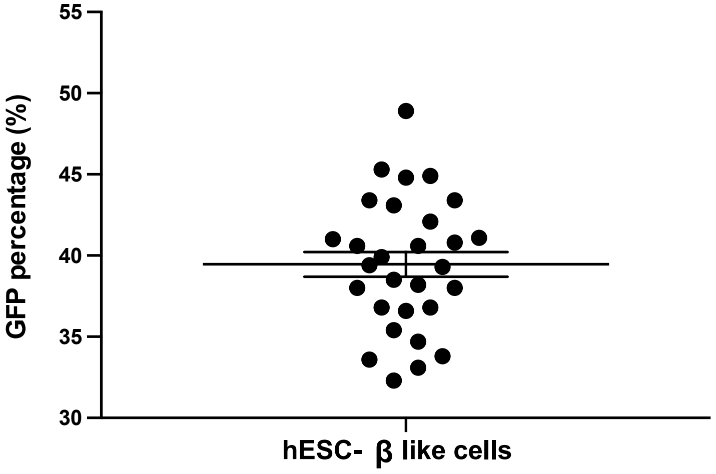
55
50
45
40
35
30
GFP percentage (%)
hESC- β like cells

B

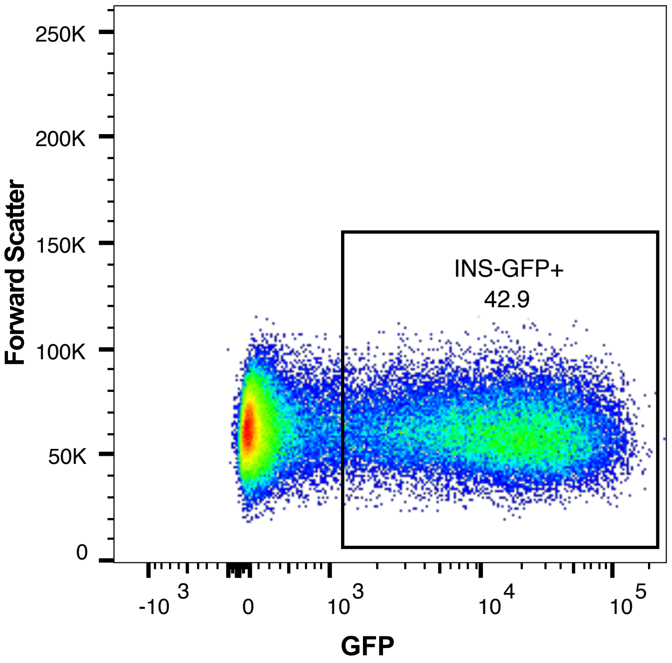
250K
200K
150K
100K
50K
0
Forward Scatter
INS-GFP+
42.9
-10 3
0
10 3
10 4
10 5
GFP

Figure S1. Related to Figure 1. Generation of hESC-β like cells that are INS-GFP+ *in vitro*.

A. The percentage of cells that INS-GFP positive produced at day 20 of differentiation protocol (hESC β-like cells). n=29 independent samples.
B. The representative FACS plot for data shown in A.

Figure S2

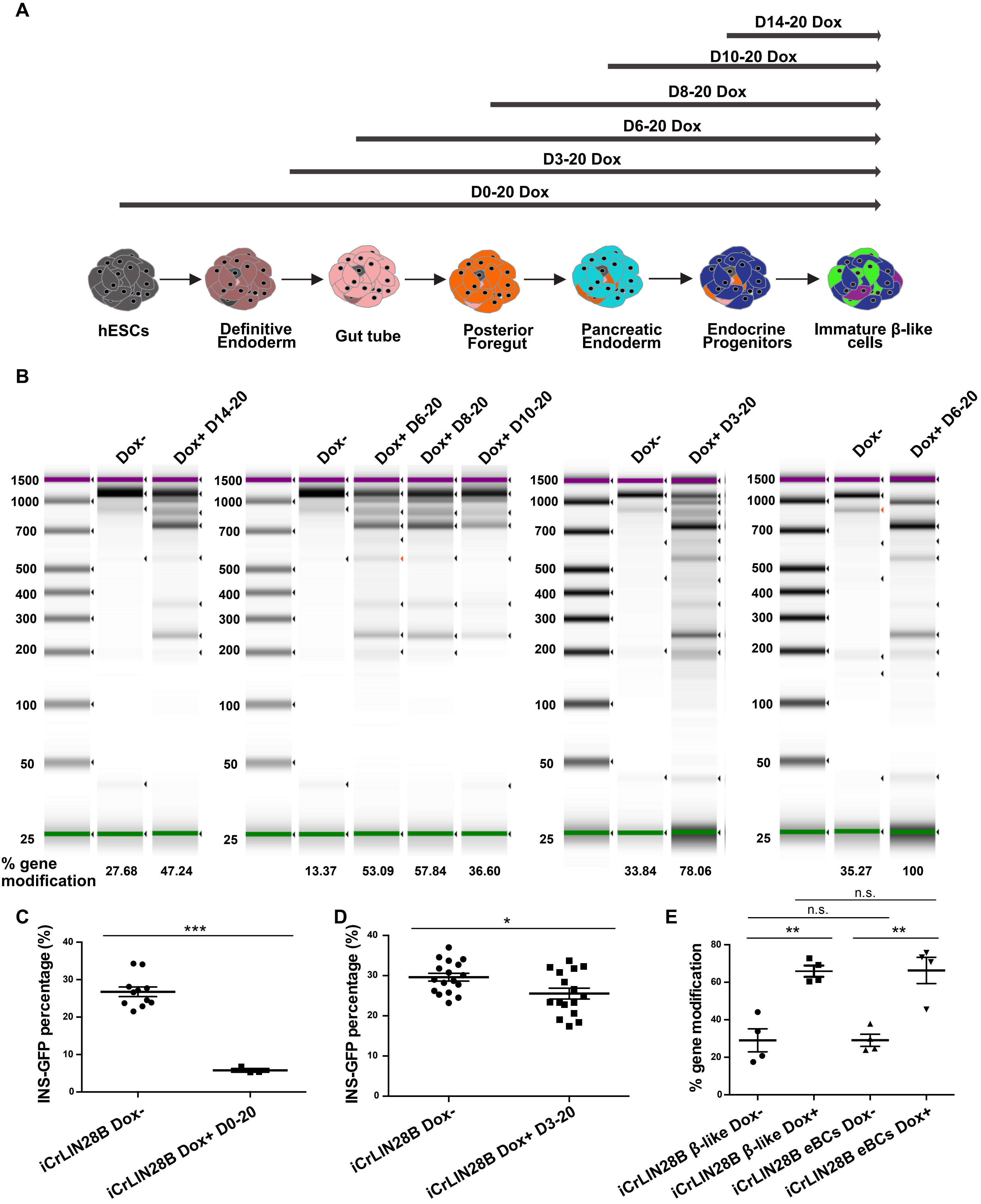
A
D14-20 Dox
D10-20 Dox
D8-20 Dox
D6-20 Dox
D3-20 Dox
D0-20 Dox
hESCs
Definitive Endoderm
Gut tube
Posterior Foregut
Pancreatic Endoderm
Endocrine Progenitors
Immature β-like cells
B
Dox-
Dox+ D14-20
Dox-
Dox+ D6-20
Dox+ D8-20
Dox+ D10-20
Dox-
Dox+ D3-20
Dox-
Dox+ D6-20
1500
1000
700
500
400
300
200
100
50
25
% gene modification
27.68
47.24
13.37
53.09
57.84
36.60
33.84
78.06
35.27
100
C
***
INS-GFP percentage (%)
iCrLIN28B Dox-
iCrLIN28B Dox+ D0-20
D
*
INS-GFP percentage (%)
iCrLIN28B Dox-
iCrLIN28B Dox+ D3-20
E
n.s.
n.s.
**
**
% gene modification
iCrLIN28B β-like Dox-
iCrLIN28B β-like Dox+
iCrLIN28B eBCs Dox-
iCrLIN28B eBCs Dox+

Figure S2. Related to Figure 3. Optimizing CRISPR-CAS9 conditions for LIN28B deletion.

A. Schematic outlining doxycycline treatment. Doxycycline was introduced at different times during the differentiation process and then kept on until the end of differentiation protocol.
B. T7EI assay in iCrLIN28B D20 spheres with doxycycline treatment from different time points and without doxycycline treatment.
C. iCrLIN28B hESC β-like cell percentage was dramatically down-regulated upon doxycycline treatment from D0 to D20. n=12 independent samples for Dox-, n=3 independent samples for Dox+. Statistical significance was calculated using unpaired two-tailed *t*-test. *p<0.05, **p<0.01, ***p<0.001, and n.s., not significant.
D. iCrLIN28B hESC β-like cell percentage was slightly down-regulated upon doxycycline treatment from D3 to D20. Statistical significance was calculated using unpaired two-tailed *t*-test. *p<0.05, **p<0.01, ***p<0.001, and n.s., not significant.
E. T7EI assay quantification in iCrLIN28B D20 spheres and iCrLIN28B eBCs. Doxycycline treatment from D3 to D20. The indel formation showed no significant difference between iCrLIN28B D20 spheres and iCrLIN28B D27 eBCs. n=4 independent samples for iCrLIN28B β-like Dox-, n=4 independent samples for iCrLIN28B β-like Dox+, n=4 independent samples for iCrLIN28B eBCs Dox-, n=4 independent experiments for iCrLIN28B eBCs Dox+. Statistical significance was calculated using unpaired two-tailed *t*-test. *p<0.05, **p<0.01, ***p<0.001, and n.s., not significant.

Figure S3

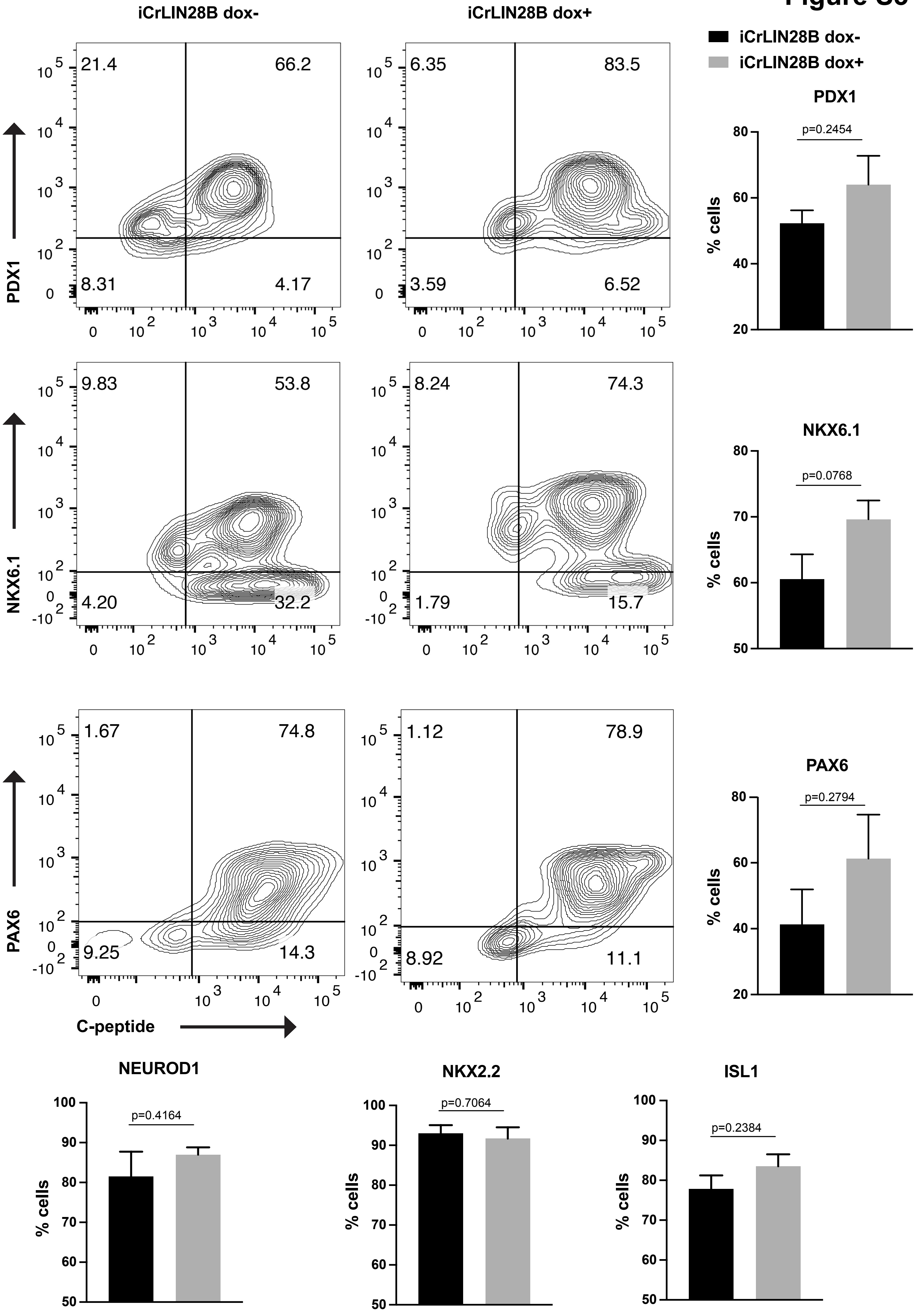
iCrLIN28B dox-
iCrLIN28B dox+
iCrLIN28B dox-
iCrLIN28B dox+
21.4
66.2
8.31
4.17
6.35
83.5
3.59
6.52
PDX1
p=0.2454
% cells
9.83
53.8
4.20
32.2
8.24
74.3
1.79
15.7
NKX6.1
p=0.0768
1.67
74.8
9.25
14.3
1.12
78.9
8.92
11.1
PAX6
p=0.2794
C-peptide
NEUROD1
p=0.4164
NKX2.2
p=0.7064
ISL1
p=0.2384

Supplemental Data File 1: Let-7 overexpression cassette in iLET-7

The sequence of let-7 overexpression cassette in iLET-7. miRNAs that are excised by Dicer are in red, pre-miR-21 loop in blue.

Supplemental Tables:

Table S1: Human islets vs hESC-β like cells differential miRNA expression, Related to Figure 1.

Table S2: Post Tx hESC-β cells vs hESC-β like cells differential miRNA expression, Related to Figure 1.

Table S3: hESC-β like cells vs human islets differential mRNA expression, Related to Figure Figure 2.

Table S4: hESC-β like cells vs Post Tx hESC-β cells differential mRNA expression, Related to Figure 2.

Table S5: Raw values for static GSIS assays, Related to Figure 3.

Table S6: ICrLIN28B DOX– vs. iCrLIN28 DOX+ differential mRNA expression, Related to Figure 3.

SUPPLEMENTAL EXPERIMENTAL PROCEDURES

Cell Culture and hESC-β cell differentiation

Undifferentiated INS-GFP hES cells (Micallef et al., 2012) were maintained on CF-1 mouse embryo fibroblast feeder layers in hESC media as described (Russ et al., 2015). CF-1 mouse embryo fibroblast feeder layers were prepared as described previously (Czechanski et al., 2014). Suspension-based *in vitro* differentiations were performed as described (Russ et al., 2015). D0: hESC media without FGF2. D1: RPMI (Gibco) with 0.2% FBS, 1:5,000 ITS (Gibco), 100 ng/ml activin A, and 50 ng/ml WNT3a (R&D Systems). D2: RPMI with 0.2% FBS, 1:2,000 ITS, and 100 ng/ml activin A; D3:RPMI with 0.2% FBS, 1:1,000 ITS, 2.5 µM TGFbi IV (CalBioChem), and 25 ng/ml KGF (R&D Systems); D4-5: RPMI with 0.4% FBS, 1:1,000 ITS, and 25 ng/ml KGF; D6-7: DMEM (Gibco) with 25 mM glucose containing 1:100 B27 (Gibco), 3 nM TTNPB (Sigma); D8: DMEM with 25 mM glucose containing 1:100 B27, 3 nM TTNPB, and 50 ng/ml EGF (R&D Systems); D9-11: DMEM with 25mM glucose containing 1:100 B27 (Stemcell Technologies), 50ng/ml EGF (Peprotech) and 50ng/ml KGF (Peprotech). D12-20: DMEM with 25nM glucose supplemented with 1:100 GlutaMax (Gibco), 1:100 Non-essential Amino Acid (Sigma), 1:100 B27, 10ug/ml Heparin (Sigma), 10uM $ZnSO_4$ (Sigma), 1mM N-Cysteine (Sigma), 10uM ALK5 inhibitor II (Axxora), 1uM γ-secretase inhibitor XX (Millipore), 1uM 3,3',5-Triiodo-L-thyronine sodium salt (Sigma), 500uM Vitamin C (Sigma), and 500nM LDN-193189 (Stemgent). To produce eBCs (Nair et al., 2019), D20 hESC β-like cells were sorted for INS-GFP expression as described below. The sorted cells were collected and distributed into Aggrewell 400 plates (Stemcell Technologies) with 1 million cells per well to re-aggregate into spheres and cultured further for *in vitro* maturation. The spheres were cultured in CMRL (Gibco) supplemented with 1:100 GlutaMax, 1:100 Non-essential Amino Acid, 10% FBS (Gibco), 10ug/ml Heparin, 10uM $ZnSO_4$, 1mM N-Cysteine, 10uM ALK5 inhibitor II, and 1uM 3,3',5-Triiodo-L-thyronine sodium salt (Nair et al., 2019). On day 27, the resulting enriched beta clusters (eBCs) were collected for downstream experiments. Human islets were from the UCSF Islets and Cellular Production Facility.

Mice

NOD.Cg-Prkdcscid Il2rgtm1Wjl/SzJ mice (NSG) were obtained from Jackson Laboratories. Mice

used in this study were maintained according to protocols approved by the University of California, San Francisco
Committee on Laboratory Animal Resource Center. Mouse kidney capsule grafts have been described previously (Russ et al., 2015).

Flow cytometry

For RNA-seq, GFP+ cell counts and reaggregation experiments, hESC β-like day 20 spheres were collected and allowed to settle by gravity. Clusters were washed once with DPBS (Sigma) and dissociated by lightly tapping the tube and gentle pipetted twice after 8 min incubation in Accumax (Millipore). Cell suspension was filtered and sorted on FACSAriaII (BD Bioscience). Dead cells were excluded by DAPI (Invitrogen) staining. For intracellular staining of transcription factors, clusters were dissociated, fixed, permeabilized and stained for various intracellular markers for analysis on LSRFortessa X20 Dual, as described previously(Nair et al., 2019). Data were analyzed with FlowJo software. Cells obtained from day 5 of the differentiation protocol were used as negative controls as they do not express beta cell markers. Anti-human C-peptide antibodies were conjugated in-house using the Molecular Probes Antibody Labeling Kits according to manufacturer's instructions. Antibody details are listed below.

Antibody	Manufacturer	Dilution	Catalog #
Human PAX6-Alexa 647	BD Bioscience	1:50	562249
Islet-1- PE	BD Bioscience	1:50	562547
NKX6.1- Alexa 647	BD Bioscience	1:50	563338
NKX2.2-PE	BD Bioscience	1:50	564730
NeuroD1- Alexa 647	BD Bioscience	1:50	563566
PDX1-PE	BD Bioscience	1:50	562161
C-peptide-488	Chemicon (Mouse antibody) conjugated in house	1:200	C-PEP-01

Cell lines

The INS-GFP MEL-1 line was kindly provided by Ed Stanley (Micallef et al., 2012). The iCrLIN28B line is an INS-GFP MEL-1 line modified with a doxycycline inducible Cas9 and U6 expressed guide RNAs directed toward either end of exon 3 (Fig. 3A). Specifically, a cassette

carrying TRE-Cas9, two U6-gRNAs, and t2A-PURO was targeted to one hAAVS1 allele, while a CAG-M2rtTA, t2A-NEO cassette was targeted to the second hAAVS1 using TALEN driven recombineering as previously described (Gonzalez et al., 2014). The gRNA sequences were: AGAAAATCCGAAGATTTAGG and CTACAGAAAAGAAAACCAAAGGG.. The iLET-7 line is an INS-GFP MEL-1 line modified with doxycycline inducible let-7a/f/b overexpression cassette. The cassette consisted of 3 repeats the pre-let7a/f and pre-let7b hairpins with spacers in between (Fig. 4A). The loop regions of the pre-let-7f and pre-let7b hairpins were replaced with the pre-miR-21 loop to block LIN28A/B recognition and suppression of let-7 biogenesis (Piskounova et al., 2011). The let-7a loop is already resistant to LIN28A/B regulation and thus was left intact (Triboulet et al., 2015). The cassette was placed downstream of a TRE and targeted to AAVS1 locus as described above. Doxycycline was added at a final concentration of 2ug/ml when needed.

Small RNA-seq and RNA-seq

Total RNA was extracted with micro RNeasy kit (Qiagen), treated with DNase I kit (Qiagen) and quantified with Qubit (Invitrogen). Bioanalyzer (Agilent) was employed to control RNA quality. Small RNA-seq libraries were made as previously described (Hafner et al., 2012). RNA-seq libraries were made by using Smart-Seq v4 Ultra Low Input RNA Kit for Sequencing (Takara) and Nextera XT DNA Library Preparation kit (Illumina) thereafter.

Small RNA-seq and RNA-seq data analysis

For RNA-Seq analysis, the data were preprocessed using Kallisto (Bray et al., 2016) and index to Gencode Version 24. For the miRNA-Seq analysis data were preprocessed using CutAdapt v1.8 (DOI:10.14806/ej.17.1.200) to demultiplex and trim adapters, sequences were then aligned using Bowtie v1.1.2 (-n 0 -l 18 –best) to a hairpin genome downloaded from miRbase (Langmead et al., 2009). Differential expression analysis was performed using in R using the Limma-Voom analysis (Liu et al., 2015; Ritchie et al., 2015). Cutoffs for significance were set at an adjusted $p < 0.05$. Plots were generated using the tidyverse and the ggplot2 R package. Let-7 targets were obtained from the TargetScan Release 7.1:June 2016 let-7-5p/98-5p list. GSEA analysis was performed using the current release (16-Jul-2018) from http://www.gsea-msigdb.org/gsea/index.jsp (Mootha et al., 2003; Subramanian et al., 2005).The GseaPreranked tool was used on the log-fold change

ranked miRNAs. The gene sets analyzed were the juvenile vs. adult beta enriched genes from (Arda et al., 2016).

qRT-PCR

Total RNA was extracted with micro RNeasy kit (Qiagen), treated with DNase I kit (Qiagen) and reverse-transcribed using SuperScript III kit (Invitrogen) as per manufacturer's instructions. mRNA qPCR primer sequences are listed below.

Gene	Forward primers	Reverse primers
GAPDH	TGCACCACCAACTGCTTAGC	GGCATGGACTGTGGTCATGAG
LIN28B	AGCCCCTGTTTAGGAAGTGAAA	CACCACAGTTGTAGCATCTATCTCC
CAS9	CCGAAGAGGTCGTGAAGAAG	GCCTTATCCAGTTCGCTCAG
PDX1	AGTGGGCAGGCGGCG	CAACATGACAGCCAGCTCCA
NKX6.1	CACGAGACCCACTTTTTCCG	ACCAGACCTTGACCTGACTCT
NKX2.2	GTCCGGAGGAAGAGAACGAG	CCGTGCAGGGAGTACTGAAG
NERUROD1	ATAGACCTGCTAGCCCCTCA	TGGTCATGTTTCGATTTCCTTTGTT
PAX6	GTCCATCTTTGCTTGGGAAA	TAGCCAGGTTGCGAAGAACT
MAFA	TTGAGCGGAGAACGGTGATT	CGAAGGTGGGAACGGAGAAC
SOX9	CTCTGGAGACTTCTGAACGAGAG	CCTTGAAGATGGCGTTGGGG
ISL1	GGATTTGGAATGGCATGCGG	CATTTGATCCCGTACAACCTGA
HMGA2	ACCCAGGGGAAGACCCAAA	CCTCTTGGCCGTTTTTCTCCA

MiRNA qRT-PCR has been described previously (Moltzahn et al., 2011). The primers and probes for miRNA qRT-PCR are listed below. qRT-PCRs were performed on ABI 7900 system.

miRNA	Stem-loop reverse primers	Forward primers	Dual-labeled Probes
**hsa-let-7a**	CTCAACTGGTGTCGTGGAGTCGGCAATTCAGTTGAGAACTATAC	ACACTCCAGCTGGGTGAGGTAGTAGGTTGT	/56-FAM/TTCAGTTGAGAACTATAC/3IABLFQ/
**hsa-let-7b**	CTCAACTGGTGTCGTGGAGTCGGCAATTCAGTTGAGAACCACAC	ACACTCCAGCTGGGTGAGGTAGTAGGTTGT	/56-FAM/TTCAGTTGAGAACCACAC/3IABLFQ/
**hsa-let-7c**	CTCAACTGGTGTCGTGGAGTCGGCAATTCAGTTGAGAACCATAC	ACACTCCAGCTGGGTGAGGTAGTAGGTTGT	/56-FAM/TTCAGTTGAGAACCATAC/3IABLFQ/
**hsa-let-7d**	CTCAACTGGTGTCGTGGAGTCGGCAATTCAGTTGAGAACTATGC	ACACTCCAGCTGGGAGAGGTAGTAGGTTGC	/56-FAM/TTCAGTTGAGAACTATGC/3IABLFQ/
**hsa-let-7f**	CTCAACTGGTGTCGTGGAGTCGGCAATTCAGTTGAGAACTATAC	ACACTCCAGCTGGGTGAGGTAGTAGATTGT	/56-FAM/TTCAGTTGAGAACTATAC/3IABLFQ/
**hsa-let-7g**	CTCAACTGGTGTCGTGGAGTCGGCAATTCAGTTGAGAACTGTAC	ACACTCCAGCTGGGTGAGGTAGTAGTTTGT	/56-FAM/TTCAGTTGAGAACTGTAC/3IABLFQ/
**Snord15**	CTCAACTGGTGTCGTGGAGTCGGCAATTCAGTTGAGCCTTCTCA	ACACTCCAGCTGGGAGAGGCATTTGTCTGA	/56-FAM/TTCAGTTGAGCCTTCTCA /3IABKFQ/

Western Blots

For total protein extraction, cells/clusters were lysed with RIPA buffer (Thermo Scientific) with protease inhibitors (Roche) on ice. The supernatant was collected for Western blots. Proteins were quantified with BCA protein assay kit (Thermofisher). The proteins were resolved on 4-15% Mini-PROTEAN TGX gels (Bio-rad). Approximately 40ug protein were loaded per lane. The following antibodies were used for blotting: 1:1000 LIN28B (Cell Signaling Technology #4196), 1:5000 GAPDH (Santa Cruz Biotechnology sc-47724). Primary antibody incubation was performed at 4°C overnight. Secondary antibodies (LI-COR) were used at a concentration of 1:10,000. Imaging was performed using an Odyssey LICOR scanner. Quantification was performed using ImageJ software. Antibody details are listed below.

Antibody	Manufacturer	Dilution	Catalog #
LIN28B	Cell Signaling Technology	1:1000	4196
GAPDH	Santa Cruz Biotechnology	1:5000	sc-47724

T7 Endonuclease I assay (T7EI assay)

T7EI assay was employed to assess genome modification. Genomic DNA was extracted with genomic DNA purification kit (Thermofisher) as per manufacturer's instructions. Genomic regions flanking the CRSIPR target sites were PCR amplified with *LIN28B* T7 primers (forward: AAAACTTTAGCTGGACTCTGCAT; reverse: GCTGAAGGCTCAGTTCAGTACAT). PCR products were purified with PCR purification kit (Qiagen). For T7EI assays, 200ng of purified PCR products were denatured and reannealed in NEB buffer 2 (New England Biolabs) in a total volume of 19ul using the following protocol: 95°C, 5min; 95°C-85°C at -2°C/s; 85°C-25°C at -0.1°C/s; hold at 12°C. The hybridized PCR products were then treated with 1ul of T7EI (New England Biolabs) at 37°C for 20min in 20ul final reaction volume. Products were then 1:10 diluted and analyzed with High Sensitivity D1000 ScreenTape System (Agilent) on TapeStation 2200 (Agilent) according to manufacturer's instructions. Quantification was based on TapeStation readout of peak molarity. Indel percentage was determined by the formula: %gene modification=100*(1-(1-fraction cleaved)$^{1/2}$)

Glucose stimulation insulin secretion assays

For static insulin secretion assays, hESC eBCs were equally distributed into 8-strip tubes and washed twice with Krebs-Ringer Bicarbonate buffer (KRB) containing 2.8mM glucose. Samples were incubated for half an hour in KRB containing 2.8mM glucose to allow equilibration of cells. The buffer was removed and replaced with fresh KRB containing 2.8nM glucose for 30min incubation followed by 30min incubation in KRB containing 16.7mM glucose and then another 30min incubation in KRB with 30mM KCl. After each incubation period, supernatant was collected for human C-peptide-specific ELISA with C-peptide Chemiluminesence ELISA kit (ALPCO) as per manufacturer's instructions.

For dynamic insulin secretion assays, eBCs were assayed using the perifusion system from Biorep technologies. The clusters were placed on filters in plastic chambers that were maintained at 37°C in a temperature controlled environment. Under temperature- and CO_2-controlled conditions, the clusters were perifused at 100 ul min^{-1} with Krebs-Ringer buffer (KRB). After an initial 1.5 hour long preincubation in 2.8mM glucose-KRB, alternating low (2.8mM) and high (20mM) glucose were perfused through the system. Flow-through was collected over the course of the experiment, and C-peptide levels were measured using the STELLUX® Chemi Human C-peptide ELISA kit (Alpco). For the static assays, spheres were equally distributed into 8-strip tubes and washed twice with 2.8mM glucose-KRB. Samples were incubated for half an hour in KRB containing 2.8mM glucose to allow equilibration of cells. The buffer was removed and replaced with fresh KRB containing 2.8mM glucose for 30min incubation followed by 30min incubation in KRB containing 16.7mM glucose. After each incubation period, supernatant was collected for human C-peptide-specific ELISA with the STELLUX® Chemi Human C-peptide ELISA kit (Alpco) as per manufacturer's instructions.

Statistics

Statistical tests performed for specific data sets are described in the corresponding figure legends. In brief, under the assumption of normal distribution, two-tailed unpaired *t*-tests (Student's *t*-test) were used if standard deviation (SD) was equal or two-tailed unpaired *t*-tests with Welch's correction were used if SD was unequal for pairwise comparison in Figures1D, 2G, 3C~F, 4C~F, S2C~E, S3, S4E; two-tailed paired *t*-tests were used in Figure 3B. All statistical tests were

performed in GraphPad Prism Software v7. Statistical significance of the sequencing data was calculated by the Limma package using linear modeling and empirical Bayes statistics implemented in the lmFit and eBayes functions. Statistical methods were not used to determine sample size.

Accession number: The GEO accession number for the genomic data presented is GSE108654.

Supplemental References:

Arda, H.E., Li, L., Tsai, J., Torre, E.A., Rosli, Y., Peiris, H., Spitale, R.C., Dai, C., Gu, X., Qu, K., *et al.* (2016). Age-Dependent Pancreatic Gene Regulation Reveals Mechanisms Governing Human beta Cell Function. Cell Metab *23*, 909-920.

Bray, N.L., Pimentel, H., Melsted, P., and Pachter, L. (2016). Near-optimal probabilistic RNA-seq quantification. Nat Biotechnol *34*, 525-527.

Czechanski, A., Byers, C., Greenstein, I., Schrode, N., Donahue, L.R., Hadjantonakis, A.K., and Reinholdt, L.G. (2014). Derivation and characterization of mouse embryonic stem cells from permissive and nonpermissive strains. Nat Protoc *9*, 559-574.

Gonzalez, F., Zhu, Z., Shi, Z.D., Lelli, K., Verma, N., Li, Q.V., and Huangfu, D. (2014). An iCRISPR platform for rapid, multiplexable, and inducible genome editing in human pluripotent stem cells. Cell Stem Cell *15*, 215-226.

Hafner, M., Renwick, N., Farazi, T.A., Mihailovic, A., Pena, J.T., and Tuschl, T. (2012). Barcoded cDNA library preparation for small RNA profiling by next-generation sequencing. Methods *58*, 164-170.

Langmead, B., Trapnell, C., Pop, M., and Salzberg, S.L. (2009). Ultrafast and memory-efficient alignment of short DNA sequences to the human genome. Genome Biol *10*, R25.

Liu, R., Holik, A.Z., Su, S., Jansz, N., Chen, K., Leong, H.S., Blewitt, M.E., Asselin-Labat, M.L., Smyth, G.K., and Ritchie, M.E. (2015). Why weight? Modelling sample and observational level variability improves power in RNA-seq analyses. Nucleic Acids Res *43*, e97.

Micallef, S.J., Li, X., Schiesser, J.V., Hirst, C.E., Yu, Q.C., Lim, S.M., Nostro, M.C., Elliott, D.A., Sarangi, F., Harrison, L.C., *et al.* (2012). INS(GFP/w) human embryonic stem cells facilitate isolation of in vitro derived insulin-producing cells. Diabetologia *55*, 694-706.

Moltzahn, F., Hunkapiller, N., Mir, A.A., Imbar, T., and Blelloch, R. (2011). High throughput microRNA profiling: optimized multiplex qRT-PCR at nanoliter scale on the fluidigm dynamic arrayTM IFCs. Journal of visualized experiments : JoVE.

Mootha, V.K., Lindgren, C.M., Eriksson, K.F., Subramanian, A., Sihag, S., Lehar, J., Puigserver, P., Carlsson, E., Ridderstrale, M., Laurila, E.*, et al.* (2003). PGC-1alpha-responsive genes involved in oxidative phosphorylation are coordinately downregulated in human diabetes. Nat Genet *34*, 267-273.

Nair, G.G., Liu, J.S., Russ, H.A., Tran, S., Saxton, M.S., Chen, R., Juang, C., Li, M.-l., Nguyen, V.Q., Giacometti, S.*, et al.* (2019). Recapitulating endocrine cell clustering in culture promotes maturation of human stem-cell-derived β cells. Nature cell biology *21*, 263-274.

Piskounova, E., Polytarchou, C., Thornton, J.E., LaPierre, R.J., Pothoulakis, C., Hagan, J.P., Iliopoulos, D., and Gregory, R.I. (2011). Lin28A and Lin28B inhibit let-7 microRNA biogenesis by distinct mechanisms. Cell *147*, 1066-1079.

Ritchie, M.E., Phipson, B., Wu, D., Hu, Y., Law, C.W., Shi, W., and Smyth, G.K. (2015). limma powers differential expression analyses for RNA-sequencing and microarray studies. Nucleic Acids Res *43*, e47.

Russ, H.A., Parent, A.V., Ringler, J.J., Hennings, T.G., Nair, G.G., Shveygert, M., Guo, T., Puri, S., Haataja, L., Cirulli, V.*, et al.* (2015). Controlled induction of human pancreatic progenitors produces functional beta-like cells in vitro. EMBO J *34*, 1759-1772.

Subramanian, A., Tamayo, P., Mootha, V.K., Mukherjee, S., Ebert, B.L., Gillette, M.A., Paulovich, A., Pomeroy, S.L., Golub, T.R., Lander, E.S.*, et al.* (2005). Gene set enrichment analysis: a knowledge-based approach for interpreting genome-wide expression profiles. Proc Natl Acad Sci U S A *102*, 15545-15550.

Triboulet, R., Pirouz, M., and Gregory, R.I. (2015). A Single Let-7 MicroRNA Bypasses LIN28-Mediated Repression. Cell Rep *13*, 260-266.
