# Supplementary material for: LIN28B Impairs the Transition of hESC-Derived β Cells from the Juvenile to Adult State
Source: Stem Cell Reports. 2019 Dec 26;14(1):9–20. doi: 10.1016/j.stemcr.2019.11.009 (PMC6962644; doi:10.1016/j.stemcr.2019.11.009)
Supplement: Data S1. Let-7 Overexpression Cassette in iLET-7 — The sequence of let-7 overexpression cassette in iLET-7. miRNAs that are excised by Dicer are in red, pre-miR-21 loop in blue. [file mmc2.docx]

TAGTGAACCGTCAGATCGCCTCACACAGGAAACCAGGATTACCGAGGAGGAAAAAAAGCCTTCCTGTGGTGCTCAACTGTGATTCCTTTTCACCATTCACCCTGGATGTTCTCTTCACTGTGGGATGAGGTAGTAGGTTGTATAGTTTTAGGGTCACACCCACCACTGGGAGATAACTATACAATCTACTGTCTTTCCTAACGTGATAGAAAAGTCTGCATCCAGGCGGTCTGATAGAAAGTCAGTTAACTAATTGTACAATATTTAAGATTAACTTGTCTTAAAGAGATGTAGTGCAGCACCTGTTTATGGCCTGGAAATCCCTTAATTTAGAGATCCCGTCTGTAGCCCGTACACTGGATGGGGGTGGGGAAACCTCCTGCTTCTTGTCTTATTTCTCTGTGTCAGAATAAATGTATTTCCCTATTTTGATCCATGCTGATAACCTTATGTTGAAATTCTCTTTCGAAAGAGATTGTACTTTCCATTCCAGAAGAAAACATTGCTCTATCAGAGTGAGGTAGTAGATTGTATAGTTCTGTTGAATCTCATGGCTATACAATCTATTGCCTTCCCTGAGGAGTAGACTTGCTGCATTCCCCTCTTCCATCCAGATGATATTACCCCTCAGAAGAATTAATTTTGACATTTTGTATTTACAGTGGATCCGTTAATCCTCCTGCCCAGCCCTCCTAATCTGGTGACTGAGGACAAACAGGCAGAAGCTGGTGCTAAGCGAAGGGCGGCGGGCCCTCCCGCAGTGCAAGGCCGGGCCTGGCGGGGTGAGGTAGTAGGTTGTGTGGTTCTGTTGAATCTCATGGCTATACAACCTACTGCCTTCCCTGAGGAGCCCAGTGACACGACCCCATGGGAGGGCCGCCCCCTACCTCAGTGACACGACCCCACGGGAGGGCTGCCCCCCACCTCAGTGACCTGCAATTACTTTTAAGTTCCCTTCCATCCAGTCAGATGTTTGACTCCGAGGAGGAAAAAAAGCCTTCCTGTGGTGCTCAACTGTGATTCCTTTTCACCATTCACCCTGGATGTTCTCTTCACTGTGGGATGAGGTAGTAGGTTGTATAGTTTTAGGGTCACACCCACCACTGGGAGATAACTATACAATCTACTGTCTTTCCTAACGTGATAGAAAAGTCTGCATCCAGGCGGTCTGATAGAAAGTCAGTTAACTAATTGTACAATATTTAAGATTAACTTGTCTTAAAGAGATGTAGTGCAGCACCTGTTTATGGCCTGGAAATCCCTTAATTTAGAGATCCCGTCTGTAGCCCGTACACTGGATGGGGGTGGGGAAACCTCCTGCTTCTTGTCTTATTTCTCTGTGTCAGAATAAATGTATTTCCCTATTTTGATCCATGCTGATAACCTTATGTTGAAATTCTCTTTCGAAAGAGATTGTACTTTCCATTCCAGAAGAAAACATTGCTCTATCAGAGTGAGGTAGTAGATTGTATAGTTCTGTTGAATCTCATGGCTATACAATCTATTGCCTTCCCTGAGGAGTAGACTTGCTGCATTCCCCTCTTCCATCCAGATGATATTACCCCTCAGAAGAATTAATTTTGACATTTTGTATTTACAGTGGATCCGTTAATCCTCCTGCCCAGCCCTCCTAATCTGGTGACTGAGGACAAACAGGCAGAAGCTGGTGCTAAGCGAAGGGCGGCGGGCCCTCCCGCAGTGCAAGGCCGGGCCTGGCGGGGTGAGGTAGTAGGTTGTGT
