# Supplementary material for: LIN28B Impairs the Transition of hESC-Derived β Cells from the Juvenile to Adult State
Source: Stem Cell Reports. 2019 Dec 26;14(1):9–20. doi: 10.1016/j.stemcr.2019.11.009 (PMC6962644; doi:10.1016/j.stemcr.2019.11.009)
Supplement: Document S2. Article plus Supplemental Information [file mmc9.pdf]

Stem Cell Reports
Article
OPEN ACCESS

LIN28B Impairs the Transition of hESC-Derived β Cells from the Juvenile to Adult State

Xin Zhou,[1,2,4,6] Gopika G. Nair,[3,6] Holger A. Russ,[3,5,6] Cassandra D. Belair,[1,2,6] Mei-Lan Li,[3] Mayya Shveygert,[1,2] Matthias Hebrok,[3,7,*] and Robert Blelloch[1,2,7,*]
[1]The Eli and Edythe Broad Center of Regeneration Medicine and Stem Cell Research, University of California, San Francisco, CA 94143, USA
[2]Department of Urology, University of California, San Francisco, CA 94143, USA
[3]Diabetes Center, University of California, San Francisco, CA 94143, USA
[4]Present address: State Key Laboratory of Oral Diseases & National Clinical Research Center for Oral Diseases & Other Research Platforms & Department of Pediatric Dentistry, West China Hospital of Stomatology, Sichuan University, Chengdu, Sichuan 610041, China
[5]Present address: Barbara Davis Center for Diabetes, University of Colorado, Anschutz Medical Campus, Aurora, CO 80045, USA
[6]Co-first author
[7]Co-senior author
*Correspondence: matthias.hebrok@ucsf.edu (M.H.), robert.blelloch@ucsf.edu (R.B.)
https://doi.org/10.1016/j.stemcr.2019.11.009

SUMMARY

Differentiation of human embryonic stem cells into pancreatic β cells holds great promise for the treatment of diabetes. Recent advances have led to the production of glucose-responsive insulin-secreting cells *in vitro*, but resulting cells remain less mature than their adult primary β cell counterparts. The barrier(s) to *in vitro* β cell maturation are unclear. Here, we evaluated a potential role for microRNAs. MicroRNA profiling showed high expression of let-7 family microRNAs *in vivo*, but not in *in vitro* differentiated β cells. Reduced levels of let-7 *in vitro* were associated with increased levels of the RNA binding protein LIN28B, a negative regulator of let-7 biogenesis. Ablation of LIN28B during human embryonic stem cell (hESC) differentiation toward β cells led to a more mature glucose-stimulated insulin secretion profile and the suppression of juvenile-specific genes. However, let-7 overexpression had little effect. These results uncover LIN28B as a modulator of β cell maturation *in vitro*.

INTRODUCTION

A growing number of people are suffering from diabetes worldwide (Roglic and World Health Organization, 2016). Diabetes is a disease of imbalance between blood insulin and glucose levels secondary to pancreatic islet β cell loss or impaired function (Cerf, 2013). At present, type 1 diabetic (T1D) and end-stage type 2 diabetic (T2D) patients rely on exogenous injection of insulin to control blood glucose. While life sustaining, this therapy is arduous and prone to complications as it is virtually impossible to mimic the dynamic changes in insulin production and secretion performed by endogenous β cells. Transplantation of cadaveric islet cells provides an alternative option resulting in effective glycemic control, but these cells are in limited supply making it unfeasible for broad implementation (Sneddon et al., 2018). β cells produced by the differentiation of pluripotent stem cells, both human embryonic stem cells (hESCs) and induced pluripotent stem cells (iPSCs) hold great promise in filling this gap. Recent advancements have greatly improved the production of these cells *in vitro* (Nair et al., 2019; Velazco-Cruz et al., 2019; Veres et al., 2019). However, there remain differences between *in vitro* produced cells and endogenous adult β cells in their gene expression profile and secretory capacity. Therefore, it is important both conceptually and practically to understand the barriers to *in vitro* differentiation toward mature adult β cells. Since euglycemia can be restored in diabetic mice by transplantation of stem cell-derived pancreatic progenitors or β cell populations, it is speculated that the *in vivo* environment supports further maturation of generated β cells, although the changes that occur in β cells upon transplantation have not been elucidated.

Much of the progress in β cell differentiation has been achieved by optimizing combinations of signaling peptides and chemicals that recapitulate events that occur during normal development *in vivo* (Liew, 2010; Nair and Hebrok, 2015). MicroRNAs (miRNAs) represent another type of small molecule. They exist endogenously, function by coordinating the regulation of many targets, and can have profound effects on developmental cell fate decisions (Friedman et al., 2009; Shenoy and Blelloch, 2014). The let-7 family comprises one of the evolutionarily most conserved families of miRNAs (Friedman et al., 2009). Let-7 exists in a negative feedback loop with the RNA binding proteins LIN28A and LIN28B (Shyh-Chang and Daley, 2013). Let-7 inhibits production of the LIN28 proteins, while the LIN28 proteins suppress biogenesis of Let-7. This loop forms a bistable regulatory switch in a number of cell fate decisions (Thornton and Gregory, 2012). Of note, both let-7 and LIN28 have many other targets. Let-7 miRNAs act through their many targets to generally promote differentiation and suppress growth (Kumar et al., 2008; Roush and Slack, 2008), whereas LIN28 has the

This is an open access article under the CC BY license (http://creativecommons.org/licenses/by/4.0/).

opposite effect both by inhibiting let-7 and through let-7 independent mechanisms, such as increasing translation of cell-cycle mRNAs (Tsialikas and Romer-Seibert, 2015). Here, we report an increase in let-7 and decrease in LIN28B during β cell maturation. The manipulation of LIN28B, but not let-7 levels, promoted a switch to a more mature adult-like β cell phenotype *in vitro*, uncovering a let-7 independent role for LIN28B in inhibiting β cell maturation.

RESULTS

Let-7 Expression Increases with β Cell Maturation

To determine a potential role for miRNAs in the maturation of pancreatic β cells, we performed small RNA sequencing (RNA-seq) of *in vitro* stem cell-derived, *in vivo* matured, and human cadaveric islet cells. Human *in vitro* derived β-like cells were produced from hESCs using an INS-GFP reporter hESC line (Micallef et al., 2012), where GFP expression is under the control of the endogenous insulin promoter (Figure 1A, hESC immature β-like cells) (Faleo et al., 2017; Russ et al., 2015). Typically, 39.26% ± 4.09% INS-GFP+ cells were generated (Figures S1A and S1B). The β-like cells were also transplanted under the kidney capsule of immunodeficient mice to allow for further *in vivo* maturation for 4–5 weeks (referred to as *in vivo* matured hESC β cells). As the differentiation protocol produces a heterogeneous mixture of cells, the insulin-producing cells in both *in vitro* derived cultures and *in vivo* matured grafts were isolated by their GFP expression using fluorescence-activated cell sorting before transcriptome analysis. Cadaveric human islets were used as a proxy for pancreas-derived human β cells, although these islets contain a mix of cell types (approximately 50% β cells) (Cabrera et al., 2006).

Analysis of small RNA-seq data for human islet cells versus *in vitro* derived hESC β-like cells uncovered 554 significantly differentially expressed miRNAs (adjusted p value < 0.05) (Figure 1B; Table S1). Of note, this contained a large number of let-7 family members that were highly upregulated in the islet cells relative to the *in vitro* derived cells (Figure 1B, red dots). The increase in let-7 could have been contributed by non-β cells within the cadaveric human islets. Therefore, we next analyzed small RNA-seq data from *in vivo* matured INS-GFP+ hESC β cells versus INS-GFP+ hESC β-like cells (Figures 1C; Table S2). This comparison of isogenic purified INS-GFP+ cell populations controls for genetic variation and cellular heterogeneity. Still, similar to the human islets, the *in vivo* matured β cells showed elevated expression of multiple let-7 family members relative to the β-like cells (Figure 1C, red dots).

Next, we validated the association between let-7 expression and β cell maturation using a differentiation protocol that allows for further maturation of β-like cells *in vitro,* by reaggregation and culture of purified insulin-expressing cells as organoids (Nair et al., 2019). We call the resulting *in vitro* matured cells as hESC-enriched β cell clusters (eBCs) (Figure 1A). We performed qRT-PCR for representative let-7 family members in hESC β-like cells, *in vitro* matured hESC eBCs, *in vivo* matured hESC β cells, and human islets (Figure 1D). Consistent with the sequencing data, both human islet cells and *in vivo* matured hESC β cells showed dramatically increased levels of all the let-7 family members. hESC eBCs also showed elevated levels of let-7 relative to hESC β-like cells, but the levels were below human islets and *in vivo* matured hESC β cells (Figure 1D). Together, these data show a positive association between let-7 levels and the maturation of β cells.

LIN28B Expression Is Downregulated during β Cell Maturation

Let-7 expression is regulated both transcriptionally and post-transcriptionally (Roush and Slack, 2008). Post-transcriptionally, the RNA binding proteins LIN28A&B suppress the biogenesis of mature let-7 family members (Nam et al., 2011; Piskounova et al., 2011; Viswanathan et al., 2008). In turn, let-7 itself functions to suppress hundreds of downstream mRNA targets including *LIN28*A&B (Rybak et al., 2008; Yang et al., 2010). To evaluate the impact of β cell maturation on the let-7 regulatory network of genes, we performed mRNA-seq in hESC β-like cells, *in vivo* matured hESC β cells, and human islet cells. Differential expression between human islets and hESC β-like cells showed differential expression of many transcripts (Figure 2A, blue dots, adj p < 0.05, Table S3), including elevated levels of *LIN28*B (but not *LIN28*A) in the *in vitro* cells (*LIN28*B, green dot, adj p = 4 × 10^{-5}). Surprisingly, however, analysis of high-scoring Targetscan predicted targets of let-7 showed roughly equal distribution between up- and downregulated genes (Figure 2B, red dots). Furthermore, cumulative density analysis on the fold change of let-7 targets versus all genes, showed no shift in the curve (Figure 2C). We hypothesized this may be due to a confounding effect due to presence of other islet cell types besides β cells in human islets. Therefore, we next compared the GFP-sorted populations from hESC β-like cells and *in vivo* matured hESC β cells (Figure 2D, Table S4). Fewer genes were differentially expressed between these cell types, consistent with the common origin, and hence reduced heterogeneity between the two cell populations (Figure 2D, blue dots, adj p < 0.05). Again, *LIN28*B (but not *LIN28*A) was up in hESC β-like cells (*LIN28*B, green dot, adj p = 0.044). Also, predicted targets of let-7 were distributed equally among up- and downregulated genes (Figure 2E, red dots) and were not shifted in the cumulative density plot (Figure 2F). We validated the *LIN28*B findings

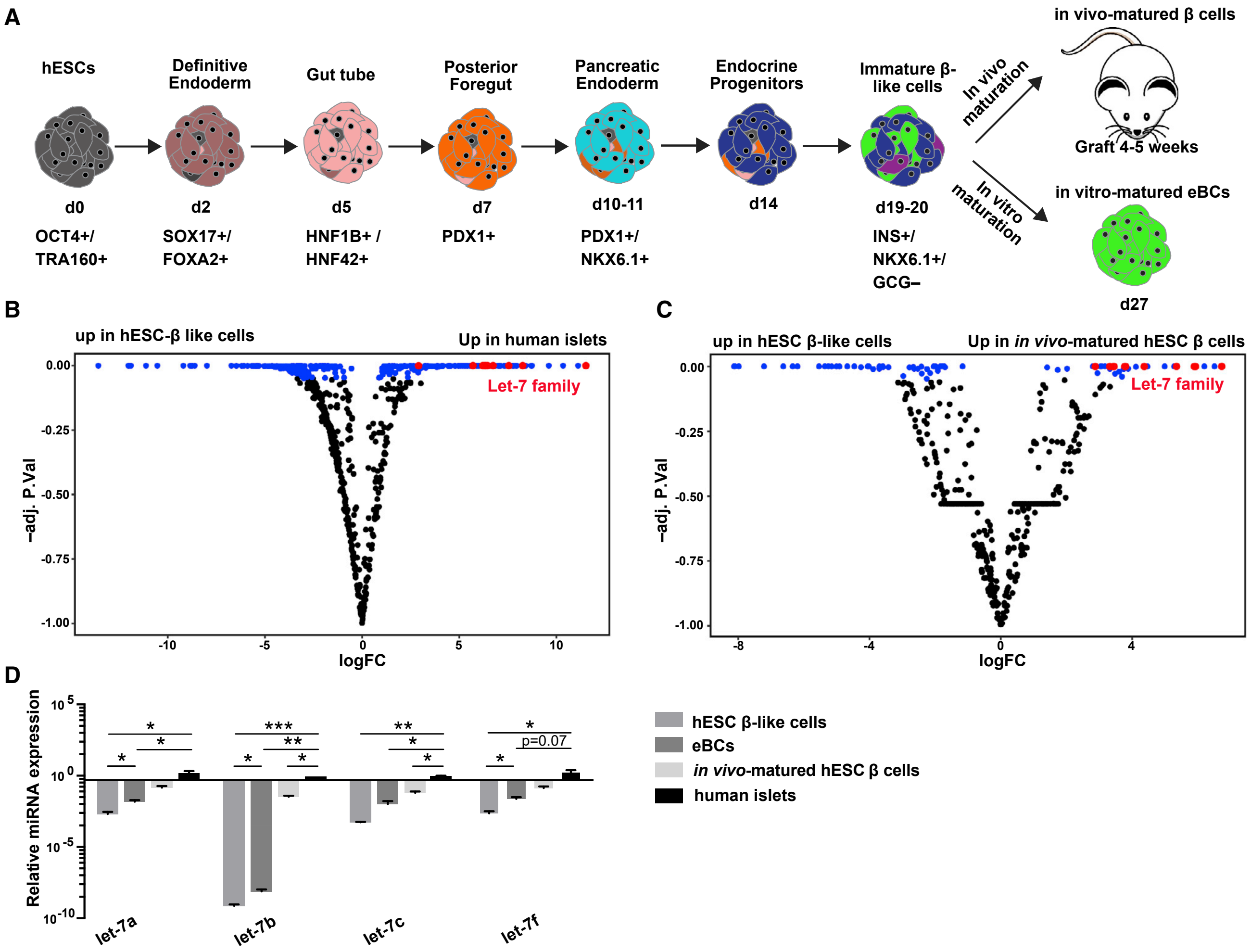

Figure 1. Let-7 Is Upregulated at Late-Stage β Cell Maturation

(A) Schematic outlining the differentiation protocol employed. *In vivo* matured β cells: β cells isolated from grafts post transplant. eBCs, enriched β clusters generated after inducing further maturation *in vitro*. Adapted from Nair et al. (2019).

(B) Volcano plot of differentially expressed miRNAs in hESC β-like cells (n = 3, independent samples) and human islets (n = 3, independent samples). Significant hits are shown in blue ($p < 0.05$). let-7 family hits are shown in red.

(C) Volcano plot of differentially expressed miRNAs in hESC β-like cells (n = 3, independent samples) and *in vivo* matured β cells (n = 3, independent samples). Significant hits are shown in blue ($p < 0.05$). let-7 family hits are shown in red.

(D) qRT-PCR verification of representative let-7 family member expression in human islets (n = 3, independent samples), hESC β-like cells (n = 4, independent samples), hESC eBCs (n = 3, independent samples), and *in vivo* matured β cells (n = 2, independent samples). Values are average ± SEM. Statistical significance was calculated using unpaired two-tailed t test. *$p < 0.05$, **$p < 0.01$, ***$p < 0.001$; n.s., not significant.

See also Figure S1 and Tables S1 and S2.

by qRT-PCR and extended them to the hESC eBCs (Figure 2G). There was a progressive reduction in *LIN28* levels when starting with hESC β-like cells, followed by hESC eBCs, *in vivo* matured hESC β cells, and finally human islet cells. The negative correlation between let-7 levels and LIN28B levels is consistent with their known negative feedback on each other (Rybak et al., 2008; Thornton and Gregory, 2012). However, the lack of enrichment of let-7 targets among the upregulated genes in hESC β-like cells suggests that let-7 downstream function may not play a major role in β cell maturation.

LIN28B Downregulation Promotes hESC β Cell Maturation

As LIN28B was up and let-7 was down in the *in vitro* derived cells (both β-like cells and eBCs) relative to *in vivo* matured hESC β cells and human islet cells, we asked if suppression of LIN28B could promote further maturation of β cells. To

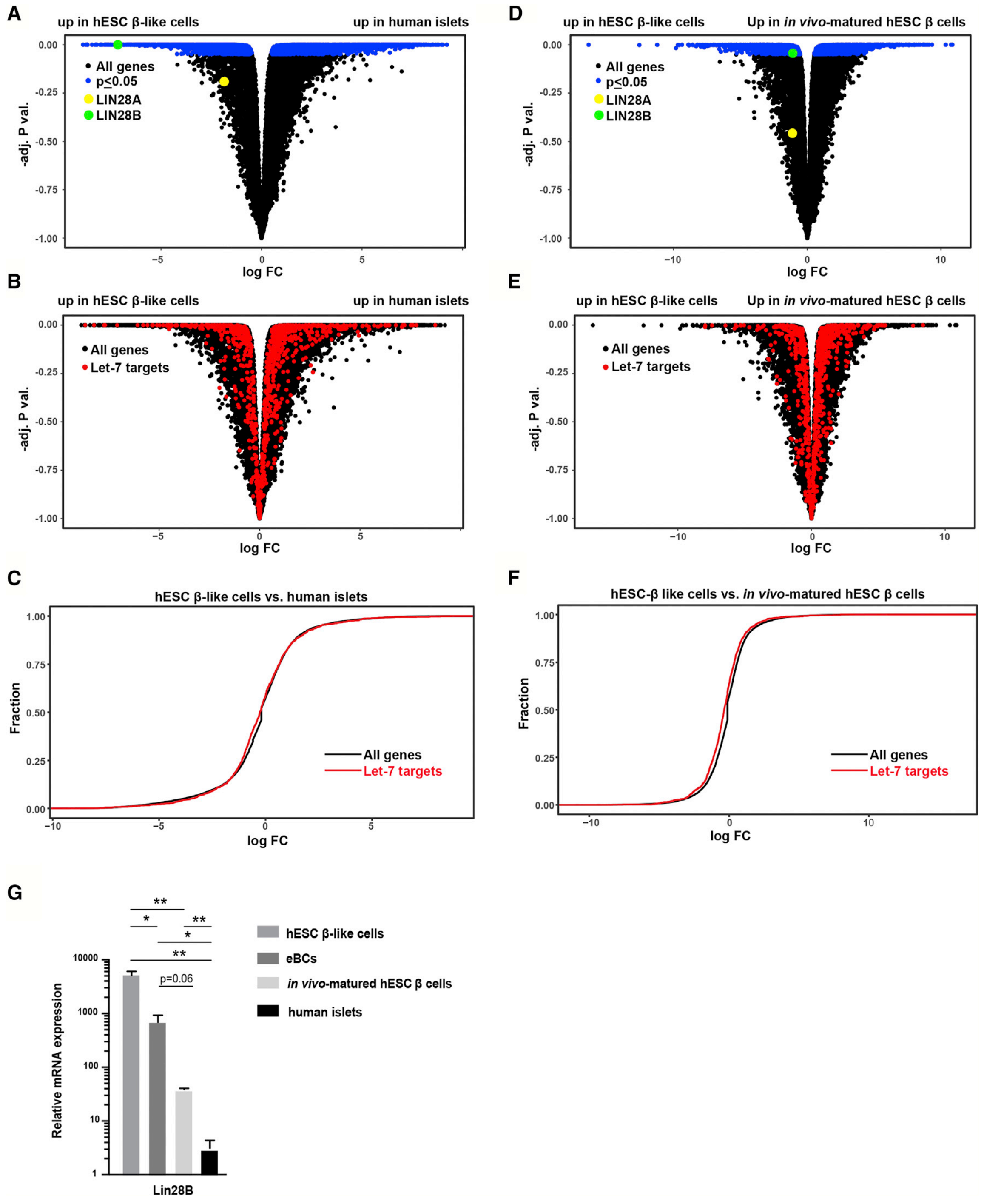

Figure 2. LIN28 Is Downregulated at Late-Stage β Cell Maturation

(A) Volcano plot of differentially expressed mRNAs in hESC β-like cells (n = 3, independent samples) and human islets (n = 3, independent samples). Significant hits are shown in blue ($p < 0.05$). LIN28A and LIN28B are highlighted in yellow and green, respectively.

(B) Same as (A), except let-7 family target genes (predicted by TargetScan) are highlighted in red.

(C) Cumulative distribution of differential expression of all expressed mRNAs and let-7 targets, predicted by TargetScan, in the human islets versus hESC β-like cells from (A).

(D) Volcano plot of differentially expressed mRNAs in hESC β-like cells (n = 3, independent samples) and *in vivo* matured β cells (n = 3, independent samples). Significant hits are shown in blue ($p < 0.05$). LIN28A and LIN28B are highlighted in yellow and green, respectively.

(legend continued on next page)

suppress LIN28B, we initially attempted knocking down LIN28 using a virally transduced shRNA against the *Lin28*b mRNA. However, transduction even of a control vector led to poor differentiation (data not shown). Therefore, we implemented a doxycycline-inducible CRISPR knockout strategy with guide RNAs on either side of exon 3 (iCrLIN28B) (Gonzalez et al., 2014) (Figure 3A, also see Experimental Procedures). Both gRNAs are constitutively expressed under the control of U6 promoters, while Cas9 protein expression depends on doxycycline exposure. Doxycycline was introduced at different times during the differentiation process and the cells were treated until the end of the differentiation protocol (Figure S2A). Addition of doxycycline at day 0 resulted in 100% indel formation, but caused poor differentiation of INS-GFP+ cells (Figures S2B and S2C). In contrast, addition at day 3 resulted in 78% indel formation with only a marginal decrease in the number of INS-GFP+ cells at day 20 of differentiation (Figure S2D). Addition of doxycycline at later time points including days 6, 8, 10, and 14 resulted in lower levels of indel formation (Figure S2B), indicating a relative resistance to the inducible CRISPR knockout at later stages of differentiation. Furthermore, cells assessed at the β-like and eBC stages that were treated with doxycyline from day 3, consistently showed 70%–80% indel formation (Figure S2E). This result was corroborated with western blot analysis showing a 50% reduction in LIN28B protein levels at β-like stage of differentiation (Figure 3B). Because addition of doxycycline starting at day 3 resulted in the greatest loss of LIN28B while retaining near normal INS-GFP+ cell numbers, further experiments were performed using this treatment regime.

In particular, we evaluated the effect of iCrLIN28B on let-7 production and β cell maturation on hESC eBCs. iCrLIN28B led to a small, albeit significant, increase in let-7 levels as determined by qRT-PCR for four representative let-7 family members (Figure 3C). To determine the impact of LIN28B depletion on β cell maturation, we first analyzed the expression of key β cell markers. qRT-PCR showed enhanced expression of a number of markers of β cells including *PDX1, NKX6.1, NKX2.2, MAFA, NEUROD1*, and *ISL1* (Figure 3D). PDX1, NKX6.1, NKX2.2, and NEUROD1 are important in the maintenance of β cell identity and function (Chao et al., 2007; Gao et al., 2014; Gu et al., 2010; Schaffer et al., 2013). MAFA regulates insulin expression and promotes the functional maturation of β cells (Artner et al., 2010; Hang and Stein, 2011). ISL1 maintains the terminal differentiation program of β cells (Ediger et al., 2017). We also conducted a protein level characterization of these β cell markers by flow cytometry (Figure S3). Percentage of cells expressing PDX1, NKX6.1, PAX6, and ISL1 trended higher in LIN28B-deleted cells than controls, although the statistical tests did not reach a p value of 0.05.

Next, we tested the impact of LIN28 depletion on β cell function using both static and dynamic glucose stimulation insulin secretion (GSIS) studies. In the static GSIS assay, the fold increase in insulin release into the media was measured following an increase in the glucose concentration from 2.8 to 16.7 mM (stimulation index). The assay showed a significant, roughly 2-fold greater increase in the stimulation index in the doxycycline-treated versus untreated iCrLIN28B eBCs (Figure 3E; Table S5). For the dynamic assay, iCrLIN28B doxycycline-treated or untreated eBCs were perfused with low- and high-glucose buffers and insulin release was measured over time. In contrast to the static assay, the dynamic perifusion assay provides a comprehensive view of β cell function including the basal, first, and second phases of insulin secretion. The perifusion assay showed higher levels of insulin secretion in both the low- and high-glucose treatments for the doxycycline-treated iCrLIN28B eBCs relative to their untreated counterparts (Figure 3F). These perifusion results are highly reminiscent of differences in the secretion profiles previously reported for adult versus juvenile primary human islet samples (Arda et al., 2016).

The Arda et al. study also measured gene expression differences in adult versus juvenile primary human islet samples by RNA-seq. We therefore performed a similar RNA-seq comparison between our doxycycline-treated and untreated iCrLIN28B hESC eBCs (Figure S4A; Table S6). To compare the gene expression changes in our experiments with theirs we performed gene set enrichment analysis (GSEA) for genes upregulated in either adult or juvenile primary human islets. Although there was no enrichment for adult upregulated genes in our doxycycline-treated samples there was a highly significant depletion of the juvenile upregulated genes (Figures 3G and S4B). Together, these data show that the reduction of LIN28B during differentiation of hESCs to β cells *in vitro* promotes their maturation with a switch from a more juvenile to a more adult-like primary human β cell phenotype.

(E) Same as (D), except let-7 family target genes (predicted by TargetScan) are highlighted in red.
(F) Cumulative distribution of differential expression of all expressed mRNAs and let-7 targets, predicted by TargetScan, in the *in vivo* matured β cells versus hESC β-like cells from (D).
(G) qRT-PCR verification of *LIN28*B expression in human islets (n = 3, independent samples), hESC β-like cells (n = 4, independent samples), hESC eBCs (n = 3, independent samples), and *in vivo* matured β cells (n = 3, independent samples). Values are average ± SEM. Statistical significance was calculated using unpaired two-tailed t test. $*p < 0.05$, $**p < 0.01$, $***p < 0.001$; n.s., not significant.

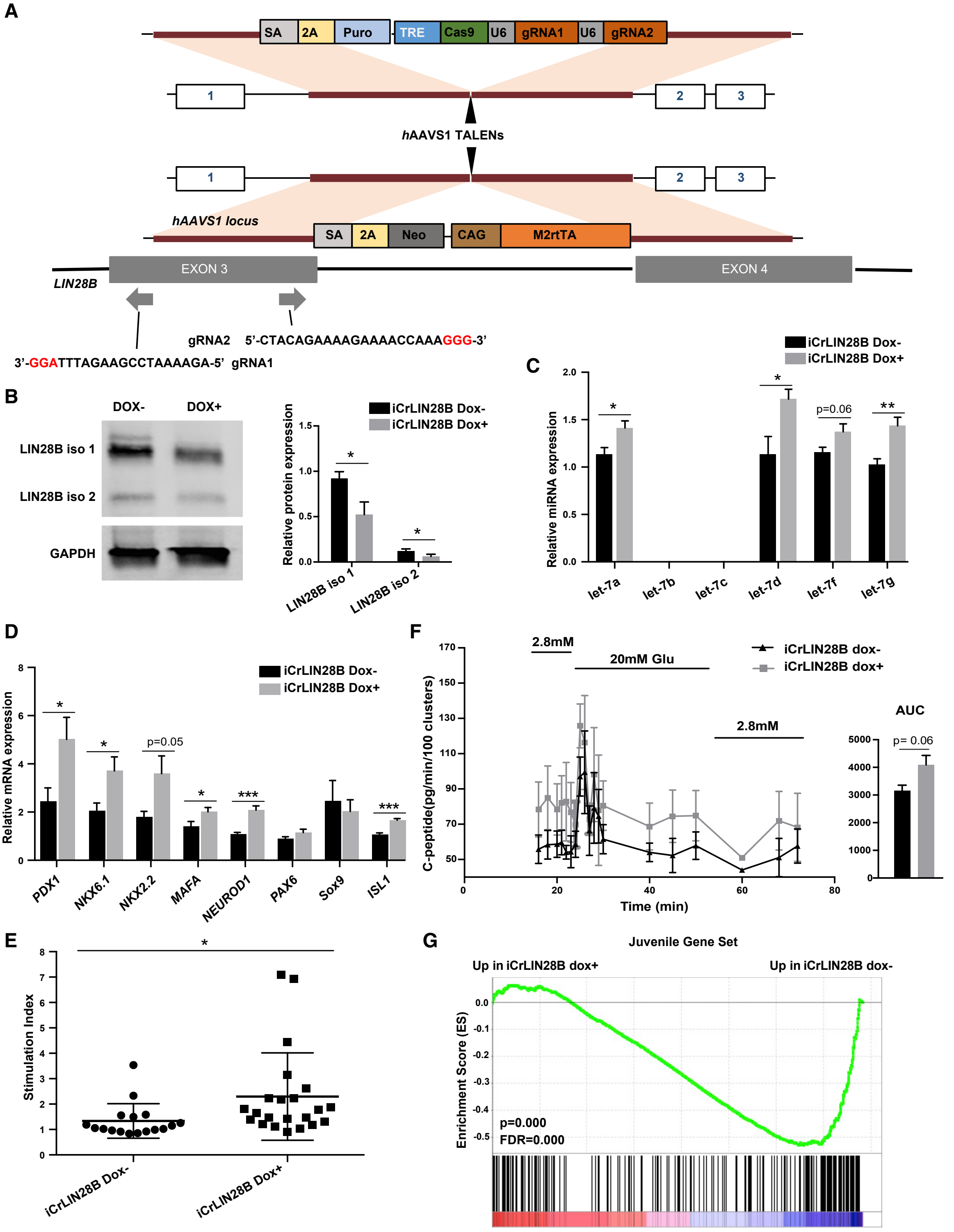

Figure 3. LIN28B Downregulation Promotes hESC-β Cell Maturation

(A) Generation of iCrLIN28B. SA, splice acceptor; 2A, self-cleaving 2A peptide; Puro, puromycin resistance gene; TRE, tetracycline response element; Cas9, Cas9 protein; U6, U6 promoter; *Neo*, neomycin resistance gene; CAG, constitutive synthetic promoter; M2rtTA, reverse tetracycline *trans*-activator sequence and protein. gRNA1 and gRNA2 were designed to target in LIN28B exon 3.

(legend continued on next page)

Let-7 Upregulation Alone Is Insufficient to Drive hESC-β Cell Maturation

Next we asked whether let-7 is acting downstream of LIN28B depletion. Induction of iCrLIN28 led to a small increase in let-7 levels (Figure 3C). Analysis of the differential expression between uninduced and induced iCrLIN28 showed no enrichment of let-7 targets among the downregulated set of genes (Figures S4C and S4D). To directly test the impact of let-7 on β cell maturation, we generated a cell line where a doxycycline-inducible let-7a/f/b transgene was targeted to the *h*AAVS1 locus (iLET-7) (Figure 4A). Doxycycline was added from day 14 to day 27 to induce let-7 at the later stages of differentiation (Figure 4B). Doxycycline treatment did not affect the percent of INS-GFP+ cells measured at day 20 (Figure S4E). qRT-PCR showed a 2- to 6-fold increase in let-7 in doxycycline-treated cells relative to no doxycycline controls, significantly higher than seen in the iCrLIN28B cells (Figure 4C, compare with 3C). These levels did not reduce LIN28B expression, but did suppress another well-known let-7 target, HMGA2 (Figure 4D). Static GSIS assays on the resulting day 27 eBCs did not show an improvement in the let-7-induced cells relative to uninduced controls, unlike Lin28B-deleted cells (Figure 4E, compare with Figures 3E; Table S5). Transcriptional markers of maturation were also mostly unchanged (Figure 4F). These data suggest that, while LIN28 acts a barrier to β cell maturation, it is likely acting independent of its role as an inhibitor of let-7 biogenesis.

DISCUSSION

Our findings uncover an important function for LIN28B during the course of hESC β cell maturation. The levels of let-7 and LIN28B correlate with the maturation status of the β cells. Let-7 family miRNAs were upregulated, while LIN28B was downregulated as the cells matured; human islets contain the highest levels of let-7 and lowest levels of LIN28B, followed by *in vivo* matured transplanted β cells, *in vitro* matured eBCs, and finally β-like cells. Furthermore, using an inducible CRISPR-Cas9 system we found that the deletion of LIN28B during the course of differentiation to eBCs improved GSIS. Interestingly, the change in the GSIS profile is reminiscent of the change previously reported when comparing adult versus juvenile primary human islets (Arda et al., 2016). Also consistent with this previous report is the observation that inducible deletion of LIN28B led to the downregulation of genes characteristic of juvenile primary human islets. In addition, the expression of a number of markers of pancreatic β cell function and maturation, including PDX1, NKX6.1, MAFA, PAX6, and ISL1, were higher upon LIN28B deletion. Together, these findings show that LIN28B suppresses maturation of hESC-derived β cells.

How LIN28B suppresses maturation is unclear. A major role of LIN28B is to inhibit the biogenesis of let-7 (Piskounova et al., 2011). However, in the context of hESC differentiation toward β cells, the reduction of LIN28B led to a very modest increase in the levels of let-7 family members. Furthermore, there was no enrichment among downregulated genes for let-7 targets and the overexpression of let-7 to levels higher than seen in LIN28B-deleted cells did not promote maturation. Therefore, LIN28 appears to be acting through let-7-independent mechanisms to enhance β cell maturation. Let-7-independent roles for LIN28 have been previously reported in different contexts (Peng et al., 2011; Tsialikas and Romer-Seibert, 2015; Xu et al., 2009; Zhang et al., 2016; Zhu et al., 2011). Importantly,

(B) Western blot analysis of LIN28B in d20 (hESC β-like) clusters generated from iCrLIN28B line plus/minus Cas9 induction with doxycycline during differentiation. Dox−, no doxycycline treatment. Dox+, doxycycline treatment from d3 to d20. Left panel: representative western blot. Right panel: quantification of four independent western blots (n = 4, independent samples, two LIN28 isoforms relative to GAPDH). Values are average ± SEM. Statistical significance was calculated using paired two-tailed t test. $^{*}p < 0.05$, $^{**}p < 0.01$, $^{***}p < 0.001$; n.s., not significant.

(C) qRT-PCR analysis of representative let-7 family members in iCrLIN28B hESC eBCs plus/minus induction of Cas9 as in (B). n = 5 independent samples for Dox−, n = 7 independent samples for Dox+. Values are average ± SEM. Statistical significance was calculated using unpaired two-tailed t test. $^{*}p < 0.05$, $^{**}p < 0.01$, $^{***}p < 0.001$; n.s., not significant.

(D) qRT-PCR analysis of selected gene expression in hESC eBCs plus/minus induction of Cas9 as in (B). Number of samples and statistics as in (C).

(E) Static glucose-stimulated insulin secretion (GSIS) of iCrLIN28B eBCs. Doxycycline treatment as in (B). n = 17 independent samples for Dox−, n = 22 independent samples, for Dox+. Values are average ± SEM. Statistical significance was calculated using unpaired two-tailed t test. $^{*}p < 0.05$, $^{**}p < 0.01$, $^{***}p < 0.001$; n.s., not significant.

(F) Dynamic GSIS of iCrLIN28B hESC eBCs in perifusion assays. Dox treatments as in (B). n = 4 independent samples for Dox−, n = 4 independent samples for Dox+. Values are average ± SEM. Area under the curve (AUC) was calculated for the secretion profiles. Error bars represent the standard error. Statistical significance was calculated using unpaired two-tailed t test.

(G) GSEA of differentially expressed genes between Dox+ versus Dox− iCrLIN28B hESC eBCs on previously identified gene set found upregulated in juvenile versus adult primary human β cells (Arda et al., 2016).

See also Figures S2, S3, and S4 and Tables S5 and S6.

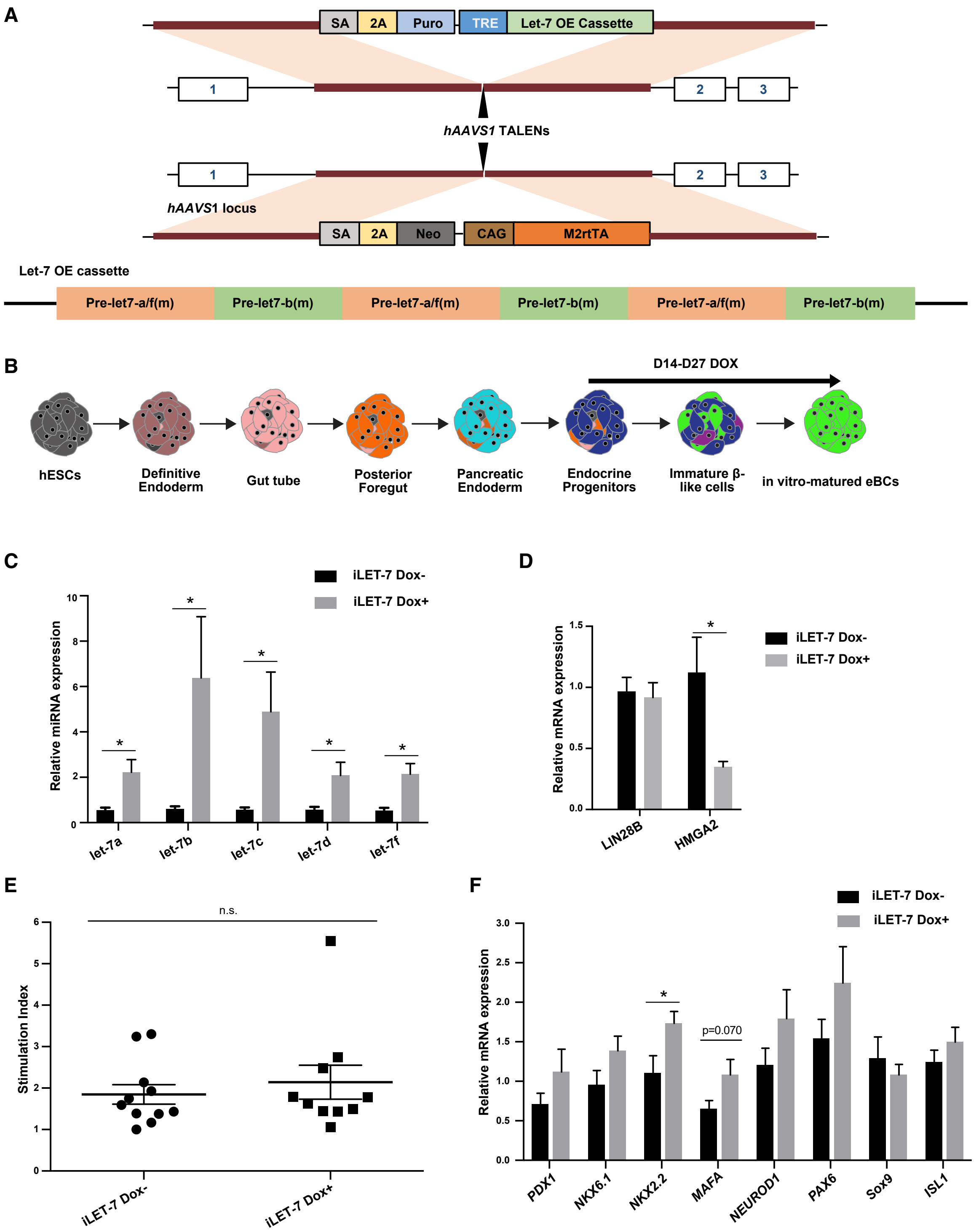

Figure 4. Let-7 Upregulation Alone Is Insufficient to Drive hESC-β Cell Maturation

(A) Generation of iLET-7 cell line. Pre-let-7a/f (m) and pre-let-7b (m) are genomic sequences for let-7a/f and let-7b precursors with LIN28 binding region mutated (see Experimental Procedures). OE, overexpression.

(B) Schematic outlining let-7 induction protocol. Doxycycline treatment was from d14 to d27. Fluorescence-activated cell sorting was performed on d20. GSIS and qRT-PCR were performed on d27.

(legend continued on next page)

our results do not rule out a role for the very high levels of let-7 seen in *in vivo* matured β cells and adult primary islets promote β cell maturation. We were not able to achieve those levels in our experiments.

A connection between LIN28 and glucose metabolism has been reported in mice. Whole-body LIN28A- and LIN28B-overexpressing transgenic animals are more sensitive to insulin and have reduced peripheral glucose levels (Zhu et al., 2011). These results compared with ours suggest opposite effects of LIN28 in the cells that produce insulin versus cells that receive the insulin signal. However, caution should be taken when comparing mouse and human β cell maturation. For example, in mouse, increased basal insulin secretion has been associated with immaturity (Blum et al., 2012; Puri et al., 2018), while in human, both basal and stimulated insulin secretion is higher in adult versus juvenile β cells (Arda et al., 2016).

Poor glucose management is associated with long-term diabetic consequences including diabetic retinopathy, nephropathy, and neuropathy (Cade, 2008). Transplantation of hESC-derived β cells holds great promise for improving glucose management and thus minimizing the negative consequences. Understanding barriers to β cell maturation has the potential to improve the functionality of transplanted cells. Our work provides insight into one such barrier whose activity could be targeted by ongoing efforts to find small-molecule inhibitors of LIN28 and its partners (Roos et al., 2016).

EXPERIMENTAL PROCEDURES

Cell Culture and hESC-β Cell Differentiation

Undifferentiated INS-GFP hES cells (Micallef et al., 2012) were maintained and differentiated into hESC-β like cells and eBCs as described previously (Nair et al., 2019; Russ et al., 2015). Human islets were from the UCSF Islets and Cellular Production Facility. The procurement and use of human islets used in the study was approved by the institutional biosafety committee at UCSF. The study is compliant with all relevant ethical regulations regarding research involving human participants, and informed consent was obtained by all participants at the isolation facility.

Mice

NOD.Cg-Prkdcscid Il2rgtm1Wjl/SzJ mice (NSG) were obtained from Jackson Laboratories. Mice used in this study were maintained according to protocols approved by the University of California, San Francisco Committee on Laboratory Animal Resource Center. Mouse kidney capsule grafts have been described previously (Russ et al., 2015).

Flow Cytometry

Stained cells were run on LSRFortessa X20 DualData and analyzed with FlowJo software. Detailed staining methods including antibodies in Supplemental Information.

iCRISPR LIN28 and iLet7

Construction of the iCRISPR line was built as described previously (Gonzalez et al., 2014). The iLET-7 strategy is shown in Figure 4A. Details in Supplemental Information.

Small RNA-Seq and RNA-Seq

Small RNA-seq libraries were made as described previously (Hafner et al., 2012). RNA-seq libraries were made by using SMART-Seq v4 Ultra Low Input RNA Kit for Sequencing (Takara) and Nextera XT DNA Library Preparation kit (Illumina) thereafter.

Small RNA-Seq and RNA-Seq Data Analysis

For RNA-seq analysis, the data were preprocessed using Kallisto (Bray et al., 2016) and index to Gencode v.24. For the miRNA-seq analysis, reads were aligned using Bowtie v1.1.2 (-n 0 -L 18 -best) to a hairpin genome downloaded from miRbase (Langmead et al., 2009). Differential expression analysis was performed using in R using the Limma-Voom analysis (Liu et al., 2015; Ritchie et al., 2015). Let-7 targets were obtained from the TargetScan Release 7.1:June 2016 let-7-5p/98-5p list. GSEA analysis was performed using the current release (July 16, 2018) from http://www.gsea-msigdb.org/gsea/index.jsp (Mootha et al., 2003; Subramanian et al., 2005).

qRT-PCR

Total RNA was extracted with RNeasy Micro Kit (QIAGEN), treated with DNase I Kit (QIAGEN), and reverse transcribed using SuperScript III Kit (Invitrogen) as per the manufacturer's instructions.

miRNA qRT-PCR has been described previously (Moltzahn et al., 2011). Primers and probes can be found in Supplemental Information.

(C) qRT-PCR analysis of representative let-7 family members in iLET-7 hESC eBCs. Doxycycline treatment as in (B). n = 6 independent samples for Dox−, n = 5 independent samples for Dox+. Values are average ± SEM. Statistical significance was calculated using unpaired two-tailed t test. *p < 0.05, **p < 0.01, ***p < 0.001; n.s., not significant.
(D) qRT-PCR analysis of *LIN28*B and *HMGA2* expression in iLET-7 hESC eBCs. Doxycycline treatment, number of replicates and statistics as in (C).
(E) Static GSIS of iLET-7. Doxycycline treatment as in (B). n = 11 independent samples for Dox−, n = 10 independent samples for Dox+. Values are average ± SEM. Statistical significance was calculated using unpaired two-tailed t test. n.s., not significant. Compare with iCrLIN28B in Figure S4E.
(F) qRT-PCR analysis of selected gene expression in iLET-7 hESC eBCs. Dox treatment, number of replicates, and statistics as in (C).
See also Figure S4.

Western Blots

Antibodies and concentrations used can be found in Supplemental Information. Imaging was performed using an Odyssey LICOR scanner and quantified using ImageJ.

T7 Endonuclease I Assay

Genomic regions flanking the CRSIPR target sites were PCR amplified. Purified PCR products were denatured and reannealed and then treated with the T7 Endonuclease I Assay (New England Biolabs). Indel percentage was determined by the formula: %gene modification = 100 × (1–(1–fraction cleaved)$^{1/2}$).

GSIS Assays

For static insulin secretion assays, cells were treated at the indicated glucose concentrations, and supernatant was collected. For dynamic insulin secretion assays, eBCs were assayed using the perifusion system from Biorep Technologies. Flow-through was collected over the course of the experiment. C-peptide levels were measured using the STELLUX Chemi Human C-peptide ELISA kit (Alpco).

ACCESSION NUMBERS

The GEO accession number for the genomic data presented is GSE108654.

SUPPLEMENTAL INFORMATION

Supplemental Information can be found online at https://doi.org/10.1016/j.stemcr.2019.11.009.

AUTHOR CONTRIBUTIONS

X.Z., M.H., and R.B. conceived of the experimental study. X.Z. performed the experiments presented in the figures except as noted below. G.G.N. developed the protocol to produce β-like cells and eBCs and conducted the experiments with end-stage cells, including perifusion assays. M.-L.L. differentiated the cells. H.A.R. developed the iCrLIN28B and iLet-7 cell lines, and performed *in vivo* transplants. M.S. and H.A.R. performed original experiments leading to premise for project. M.S. also produced sequencing libraries for Figures 1B, 1C, 2A, and 2C. C.D.B. performed all genomic analyses. X.Z., R.B., and G.G.N. wrote the paper with help from M.H.

ACKNOWLEDGMENTS

This work was funded by a grant from the Leona M. and Harry B. Helmsley Charitable Trust; United States, to R.B. Stem cell work in M.H. laboratory was supported by grants from the NIH (DK105831, DK108666); United States. G.G.N. and H.A.R. were supported by fellowship grants from the JDRF; United States.

Received: January 2, 2018
Revised: November 26, 2019
Accepted: November 27, 2019
Published: December 26, 2019

REFERENCES

Arda, H.E., Li, L., Tsai, J., Torre, E.A., Rosli, Y., Peiris, H., Spitale, R.C., Dai, C., Gu, X., Qu, K., et al. (2016). Age-dependent pancreatic gene regulation reveals mechanisms governing human beta cell function. Cell Metab. *23*, 909–920.

Artner, I., Hang, Y., Mazur, M., Yamamoto, T., Guo, M., Lindner, J., Magnuson, M.A., and Stein, R. (2010). MafA and MafB regulate genes critical to beta-cells in a unique temporal manner. Diabetes *59*, 2530–2539.

Blum, B., Hrvatin, S., Schuetz, C., Bonal, C., Rezania, A., and Melton, D.A. (2012). Functional beta-cell maturation is marked by an increased glucose threshold and by expression of urocortin 3. Nat. Biotechnol. *30*, 261–264.

Bray, N.L., Pimentel, H., Melsted, P., and Pachter, L. (2016). Near-optimal probabilistic RNA-seq quantification. Nat. Biotechnol. *34*, 525–527.

Cabrera, O., Berman, D.M., Kenyon, N.S., Ricordi, C., Berggren, P.O., and Caicedo, A. (2006). The unique cytoarchitecture of human pancreatic islets has implications for islet cell function. Proc. Natl. Acad. Sci. U S A *103*, 2334–2339.

Cade, W.T. (2008). Diabetes-related microvascular and macrovascular diseases in the physical therapy setting. Phys. Ther. *88*, 1322–1335.

Cerf, M.E. (2013). Beta cell dysfunction and insulin resistance. Front. Endocrinol. (Lausanne) *4*, 37.

Chao, C.S., Loomis, Z.L., Lee, J.E., and Sussel, L. (2007). Genetic identification of a novel NeuroD1 function in the early differentiation of islet alpha, PP and epsilon cells. Dev. Biol. *312*, 523–532.

Ediger, B.N., Lim, H.W., Juliana, C., Groff, D.N., Williams, L.T., Dominguez, G., Liu, J.H., Taylor, B.L., Walp, E.R., Kameswaran, V., et al. (2017). LIM domain-binding 1 maintains the terminally differentiated state of pancreatic beta cells. J. Clin. Invest. *127*, 215–229.

Faleo, G., Russ, H.A., Wisel, S., Parent, A.V., Nguyen, V., Nair, G.G., Freise, J.E., Villanueva, K.E., Szot, G.L., Hebrok, M., et al. (2017). Mitigating ischemic injury of stem cell-derived insulin-producing cells after transplant. Stem Cell Reports *9*, 807–819.

Friedman, R.C., Farh, K.K., Burge, C.B., and Bartel, D.P. (2009). Most mammalian mRNAs are conserved targets of microRNAs. Genome Res. *19*, 92–105.

Gao, T., McKenna, B., Li, C., Reichert, M., Nguyen, J., Singh, T., Yang, C., Pannikar, A., Doliba, N., Zhang, T., et al. (2014). Pdx1 maintains beta cell identity and function by repressing an alpha cell program. Cell Metab. *19*, 259–271.

Gonzalez, F., Zhu, Z., Shi, Z.D., Lelli, K., Verma, N., Li, Q.V., and Huangfu, D. (2014). An iCRISPR platform for rapid, multiplexable, and inducible genome editing in human pluripotent stem cells. Cell Stem Cell *15*, 215–226.

Gu, C., Stein, G.H., Pan, N., Goebbels, S., Hornberg, H., Nave, K.A., Herrera, P., White, P., Kaestner, K.H., Sussel, L., et al. (2010). Pancreatic beta cells require NeuroD to achieve and maintain functional maturity. Cell Metab. *11*, 298–310.

Hafner, M., Renwick, N., Farazi, T.A., Mihailovic, A., Pena, J.T., and Tuschl, T. (2012). Barcoded cDNA library preparation for

small RNA profiling by next-generation sequencing. Methods *58*, 164–170.

Hang, Y., and Stein, R. (2011). MafA and MafB activity in pancreatic beta cells. Trends Endocrinol. Metab. *22*, 364–373.

Kumar, M.S., Erkeland, S.J., Pester, R.E., Chen, C.Y., Ebert, M.S., Sharp, P.A., and Jacks, T. (2008). Suppression of non-small cell lung tumor development by the let-7 microRNA family. Proc. Natl. Acad. Sci. U S A *105*, 3903–3908.

Langmead, B., Trapnell, C., Pop, M., and Salzberg, S.L. (2009). Ultrafast and memory-efficient alignment of short DNA sequences to the human genome. Genome Biol. *10*, R25.

Liew, C.G. (2010). Generation of insulin-producing cells from pluripotent stem cells: from the selection of cell sources to the optimization of protocols. Rev. Diabet. Stud. *7*, 82–92.

Liu, R., Holik, A.Z., Su, S., Jansz, N., Chen, K., Leong, H.S., Blewitt, M.E., Asselin-Labat, M.L., Smyth, G.K., and Ritchie, M.E. (2015). Why weight? Modelling sample and observational level variability improves power in RNA-seq analyses. Nucleic Acids Res. *43*, e97.

Micallef, S.J., Li, X., Schiesser, J.V., Hirst, C.E., Yu, Q.C., Lim, S.M., Nostro, M.C., Elliott, D.A., Sarangi, F., Harrison, L.C., et al. (2012). INS(GFP/w) human embryonic stem cells facilitate isolation of in vitro derived insulin-producing cells. Diabetologia *55*, 694–706.

Moltzahn, F., Hunkapiller, N., Mir, A.A., Imbar, T., and Blelloch, R. (2011). High throughput microRNA profiling: optimized multiplex qRT-PCR at nanoliter scale on the fluidigm dynamic arrayTM IFCs. J. Vis. Exp. *54*. https://doi.org/10.3791/2552.

Mootha, V.K., Lindgren, C.M., Eriksson, K.F., Subramanian, A., Sihag, S., Lehar, J., Puigserver, P., Carlsson, E., Ridderstrale, M., Laurila, E., et al. (2003). PGC-1alpha-responsive genes involved in oxidative phosphorylation are coordinately downregulated in human diabetes. Nat. Genet. *34*, 267–273.

Nair, G., and Hebrok, M. (2015). Islet formation in mice and men: lessons for the generation of functional insulin-producing β-cells from human pluripotent stem cells. Curr. Opin. Genet. Dev. *32*, 171–180.

Nair, G.G., Liu, J.S., Russ, H.A., Tran, S., Saxton, M.S., Chen, R., Juang, C., Li, M.L., Nguyen, V.Q., Giacometti, S., et al. (2019). Recapitulating endocrine cell clustering in culture promotes maturation of human stem-cell-derived beta cells. Nat. Cell Biol. *21*, 263–274.

Nam, Y., Chen, C., Gregory, R.I., Chou, J.J., and Sliz, P. (2011). Molecular basis for interaction of let-7 microRNAs with Lin28. Cell *147*, 1080–1091.

Peng, S., Chen, L.L., Lei, X.X., Yang, L., Lin, H., Carmichael, G.G., and Huang, Y. (2011). Genome-wide studies reveal that Lin28 enhances the translation of genes important for growth and survival of human embryonic stem cells. Stem Cells *29*, 496–504.

Piskounova, E., Polytarchou, C., Thornton, J.E., LaPierre, R.J., Pothoulakis, C., Hagan, J.P., Iliopoulos, D., and Gregory, R.I. (2011). Lin28A and Lin28B inhibit let-7 microRNA biogenesis by distinct mechanisms. Cell *147*, 1066–1079.

Puri, S., Roy, N., Russ, H.A., Leonhardt, L., French, E.K., Roy, R., Bengtsson, H., Scott, D.K., Stewart, A.F., and Hebrok, M. (2018). Replication confers beta cell immaturity. Nat. Commun. *9*, 485.

Ritchie, M.E., Phipson, B., Wu, D., Hu, Y., Law, C.W., Shi, W., and Smyth, G.K. (2015). limma powers differential expression analyses for RNA-sequencing and microarray studies. Nucleic Acids Res. *43*, e47.

Roglic, G.; World Health Organization (2016). Global Report on Diabetes (World Health Organization).

Roos, M., Pradere, U., Ngondo, R.P., Behera, A., Allegrini, S., Civenni, G., Zagalak, J.A., Marchand, J.R., Menzi, M., Towbin, H., et al. (2016). A small-molecule inhibitor of Lin28. ACS Chem. Biol. *11*, 2773–2781.

Roush, S., and Slack, F.J. (2008). The let-7 family of microRNAs. Trends Cell Biol. *18*, 505–516.

Russ, H.A., Parent, A.V., Ringler, J.J., Hennings, T.G., Nair, G.G., Shveygert, M., Guo, T., Puri, S., Haataja, L., Cirulli, V., et al. (2015). Controlled induction of human pancreatic progenitors produces functional beta-like cells in vitro. EMBO J. *34*, 1759–1772.

Rybak, A., Fuchs, H., Smirnova, L., Brandt, C., Pohl, E.E., Nitsch, R., and Wulczyn, F.G. (2008). A feedback loop comprising lin-28 and let-7 controls pre-let-7 maturation during neural stem-cell commitment. Nat. Cell Biol. *10*, 987–993.

Schaffer, A.E., Taylor, B.L., Benthuysen, J.R., Liu, J., Thorel, F., Yuan, W., Jiao, Y., Kaestner, K.H., Herrera, P.L., Magnuson, M.A., et al. (2013). Nkx6.1 controls a gene regulatory network required for establishing and maintaining pancreatic beta cell identity. PLoS Genet. *9*, e1003274.

Shenoy, A., and Blelloch, R.H. (2014). Regulation of microRNA function in somatic stem cell proliferation and differentiation. Nat. Rev. Mol. Cell Biol. *15*, 565–576.

Shyh-Chang, N., and Daley, G.Q. (2013). Lin28: primal regulator of growth and metabolism in stem cells. Cell Stem Cell *12*, 395–406.

Sneddon, J.B., Tang, Q., Stock, P., Bluestone, J.A., Roy, S., Desai, T., and Hebrok, M. (2018). Stem cell therapies for treating diabetes: progress and remaining challenges. Cell Stem Cell *22*, 810–823.

Subramanian, A., Tamayo, P., Mootha, V.K., Mukherjee, S., Ebert, B.L., Gillette, M.A., Paulovich, A., Pomeroy, S.L., Golub, T.R., Lander, E.S., et al. (2005). Gene set enrichment analysis: a knowledge-based approach for interpreting genome-wide expression profiles. Proc. Natl. Acad. Sci. U S A *102*, 15545–15550.

Thornton, J.E., and Gregory, R.I. (2012). How does Lin28 let-7 control development and disease? Trends Cell Biol. *22*, 474–482.

Tsialikas, J., and Romer-Seibert, J. (2015). LIN28: roles and regulation in development and beyond. Development *142*, 2397–2404.

Velazco-Cruz, L., Song, J., Maxwell, K.G., Goedegebuure, M.M., Augsornworawat, P., Hogrebe, N.J., and Millman, J.R. (2019). Acquisition of dynamic function in human stem cell-derived beta cells. Stem Cell Reports *12*, 351–365.

Veres, A., Faust, A.L., Bushnell, H.L., Engquist, E.N., Kenty, J.H., Harb, G., Poh, Y.C., Sintov, E., Gurtler, M., Pagliuca, F.W., et al. (2019). Charting cellular identity during human in vitro beta-cell differentiation. Nature *569*, 368–373.

Viswanathan, S.R., Daley, G.Q., and Gregory, R.I. (2008). Selective blockade of microRNA processing by Lin28. Science *320*, 97–100.

Xu, B., Zhang, K., and Huang, Y. (2009). Lin28 modulates cell growth and associates with a subset of cell cycle regulator mRNAs in mouse embryonic stem cells. RNA *15*, 357–361.

Yang, X., Lin, X., Zhong, X., Kaur, S., Li, N., Liang, S., Lassus, H., Wang, L., Katsaros, D., Montone, K., et al. (2010). Double-negative feedback loop between reprogramming factor LIN28 and microRNA let-7 regulates aldehyde dehydrogenase 1-positive cancer stem cells. Cancer Res. *70*, 9463–9472.

Zhang, J., Ratanasirintrawoot, S., Chandrasekaran, S., Wu, Z., Ficarro, S.B., Yu, C., Ross, C.A., Cacchiarelli, D., Xia, Q., Seligson, M., et al. (2016). LIN28 regulates stem cell metabolism and conversion to primed pluripotency. Cell Stem Cell *19*, 66–80.

Zhu, H., Shyh-Chang, N., Segre, A.V., Shinoda, G., Shah, S.P., Einhorn, W.S., Takeuchi, A., Engreitz, J.M., Hagan, J.P., Kharas, M.G., et al. (2011). The Lin28/let-7 axis regulates glucose metabolism. Cell *147*, 81–94.

Stem Cell Reports, Volume *14*

Supplemental Information

LIN28B Impairs the Transition of hESC-Derived β Cells from the Juvenile to Adult State

Xin Zhou, Gopika G. Nair, Holger A. Russ, Cassandra D. Belair, Mei-Lan Li, Mayya Shveygert, Matthias Hebrok, and Robert Blelloch

Figure S1

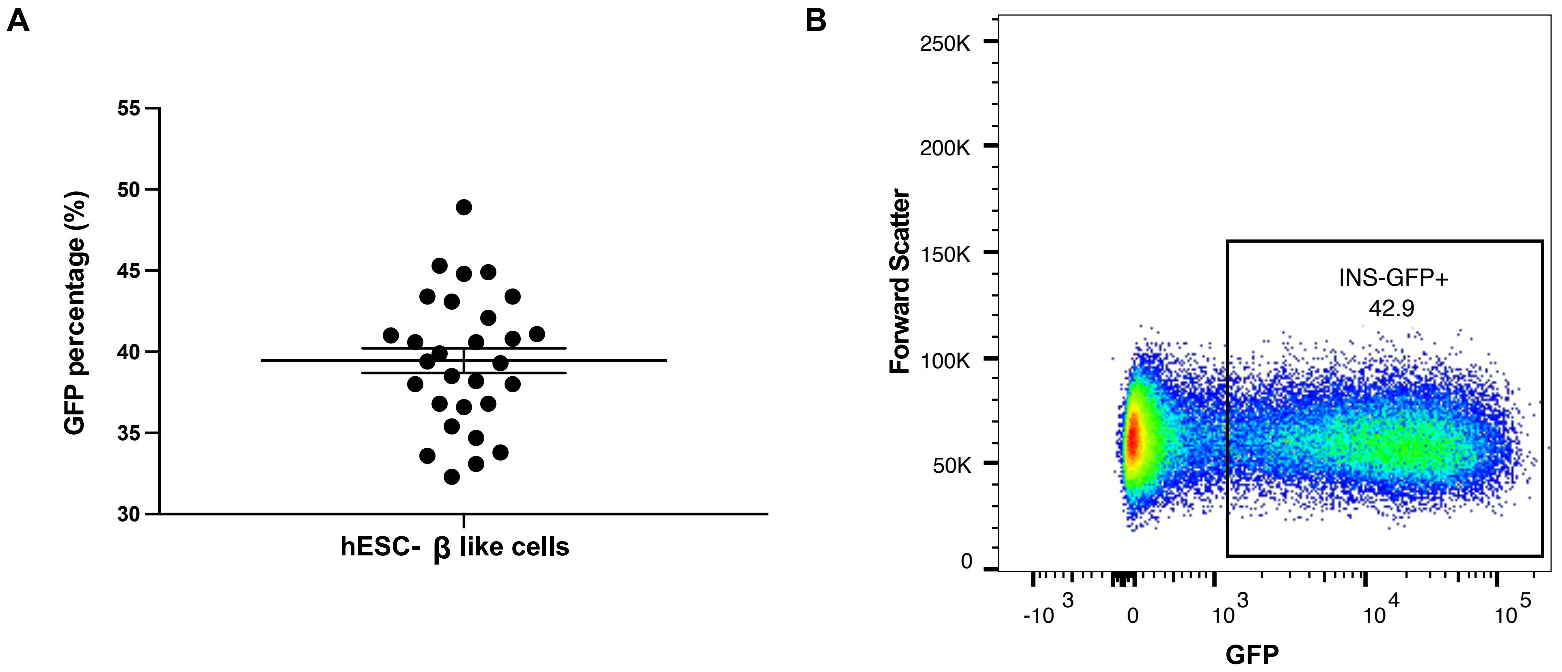
A
55
50
45
40
35
30
GFP percentage (%)
hESC- β like cells
B
250K
200K
150K
100K
50K
0
Forward Scatter
INS-GFP+
42.9
-10 3
0
10 3
10 4
10 5
GFP

Figure S1. Related to Figure 1. Generation of hESC-β like cells that are INS-GFP+ *in vitro*.

A. The percentage of cells that INS-GFP positive produced at day 20 of differentiation protocol (hESC β-like cells). n=29 independent samples.
B. The representative FACS plot for data shown in A.

Figure S2

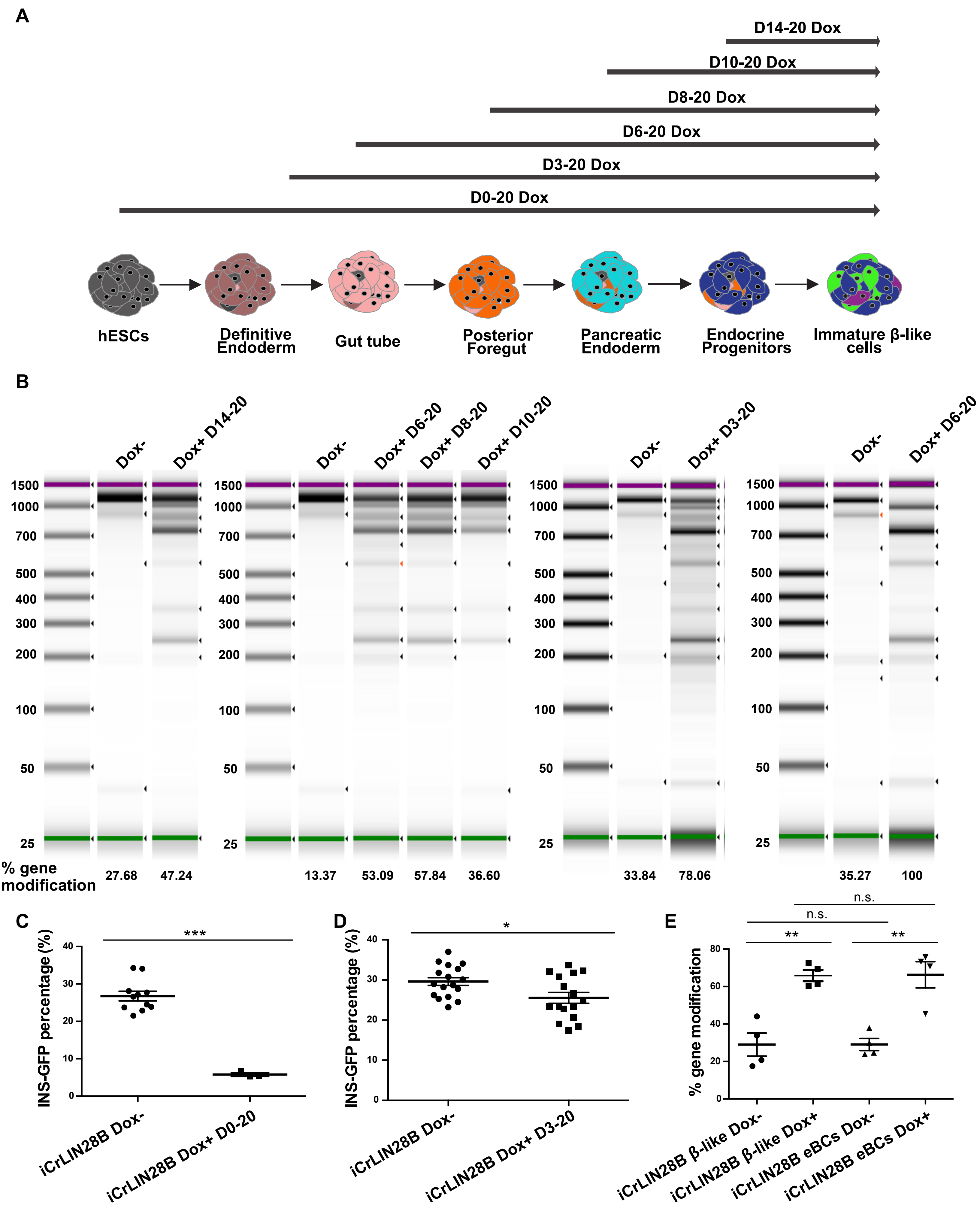

A
D14-20 Dox
D10-20 Dox
D8-20 Dox
D6-20 Dox
D3-20 Dox
D0-20 Dox
hESCs
Definitive Endoderm
Gut tube
Posterior Foregut
Pancreatic Endoderm
Endocrine Progenitors
Immature β-like cells
B
Dox-
Dox+ D14-20
Dox+ D6-20
Dox+ D8-20
Dox+ D10-20
Dox+ D3-20
1500
1000
700
500
400
300
200
100
50
25
% gene modification
27.68
47.24
13.37
53.09
57.84
36.60
33.84
78.06
35.27
100
C
***
INS-GFP percentage (%)
iCrLIN28B Dox-
iCrLIN28B Dox+ D0-20
D
*
iCrLIN28B Dox+ D3-20
E
n.s.
**
% gene modification
iCrLIN28B β-like Dox-
iCrLIN28B β-like Dox+
iCrLIN28B eBCs Dox-
iCrLIN28B eBCs Dox+

Figure S2. Related to Figure 3. Optimizing CRISPR-CAS9 conditions for LIN28B deletion.

A. Schematic outlining doxycycline treatment. Doxycycline was introduced at different times during the differentiation process and then kept on until the end of differentiation protocol.

B. T7EI assay in iCrLIN28B D20 spheres with doxycycline treatment from different time points and without doxycycline treatment.

C. iCrLIN28B hESC β-like cell percentage was dramatically down-regulated upon doxycycline treatment from D0 to D20. n=12 independent samples for Dox-, n=3 independent samples for Dox+. Statistical significance was calculated using unpaired two-tailed *t*-test. *p<0.05, **p<0.01, ***p<0.001, and n.s., not significant.

D. iCrLIN28B hESC β-like cell percentage was slightly down-regulated upon doxycycline treatment from D3 to D20. Statistical significance was calculated using unpaired two-tailed *t*-test. *p<0.05, **p<0.01, ***p<0.001, and n.s., not significant.

E. T7EI assay quantification in iCrLIN28B D20 spheres and iCrLIN28B eBCs. Doxycycline treatment from D3 to D20. The indel formation showed no significant difference between iCrLIN28B D20 spheres and iCrLIN28B D27 eBCs. n=4 independent samples for iCrLIN28B β-like Dox-, n=4 independent samples for iCrLIN28B β-like Dox+, n=4 independent samples for iCrLIN28B eBCs Dox-, n=4 independent experiments for iCrLIN28B eBCs Dox+. Statistical significance was calculated using unpaired two-tailed *t*-test. *p<0.05, **p<0.01, ***p<0.001, and n.s., not significant.

Figure S3

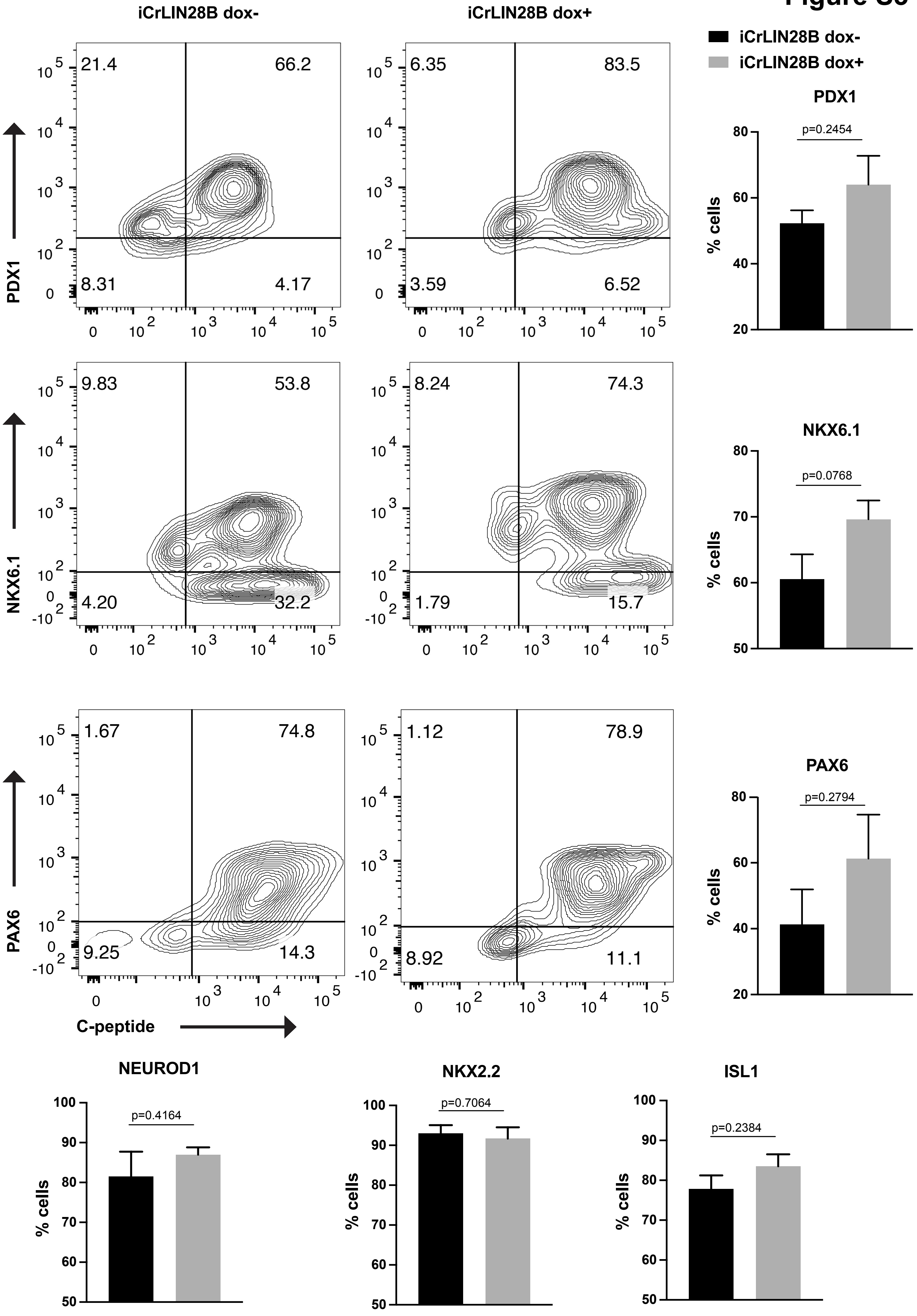
iCrLIN28B dox-
iCrLIN28B dox+
PDX1
NKX6.1
PAX6
C-peptide
21.4
66.2
8.31
4.17
6.35
83.5
3.59
6.52
9.83
53.8
4.20
32.2
8.24
74.3
1.79
15.7
1.67
74.8
9.25
14.3
1.12
78.9
8.92
11.1
p=0.2454
p=0.0768
p=0.2794
% cells
NEUROD1
NKX2.2
ISL1
p=0.4164
p=0.7064
p=0.2384

Supplemental Data File 1: Let-7 overexpression cassette in iLET-7

The sequence of let-7 overexpression cassette in iLET-7. miRNAs that are excised by Dicer are in red, pre-miR-21 loop in blue.

Supplemental Tables:

Table S1: Human islets vs hESC-β like cells differential miRNA expression, Related to Figure 1.

Table S2: Post Tx hESC-β cells vs hESC-β like cells differential miRNA expression, Related to Figure 1.

Table S3: hESC-β like cells vs human islets differential mRNA expression, Related to Figure Figure 2.

Table S4: hESC-β like cells vs Post Tx hESC-β cells differential mRNA expression, Related to Figure 2.

Table S5: Raw values for static GSIS assays, Related to Figure 3.

Table S6: ICrLIN28B DOX– vs. iCrLIN28 DOX+ differential mRNA expression, Related to Figure 3.

SUPPLEMENTAL EXPERIMENTAL PROCEDURES

Cell Culture and hESC-β cell differentiation

Undifferentiated INS-GFP hES cells (Micallef et al., 2012) were maintained on CF-1 mouse embryo fibroblast feeder layers in hESC media as described (Russ et al., 2015). CF-1 mouse embryo fibroblast feeder layers were prepared as described previously (Czechanski et al., 2014). Suspension-based *in vitro* differentiations were performed as described (Russ et al., 2015). D0: hESC media without FGF2. D1: RPMI (Gibco) with 0.2% FBS, 1:5,000 ITS (Gibco), 100 ng/ml activin A, and 50 ng/ml WNT3a (R&D Systems). D2: RPMI with 0.2% FBS, 1:2,000 ITS, and 100 ng/ml activin A; D3:RPMI with 0.2% FBS, 1:1,000 ITS, 2.5 μM TGFbi IV (CalBioChem), and 25 ng/ml KGF (R&D Systems); D4-5: RPMI with 0.4% FBS, 1:1,000 ITS, and 25 ng/ml KGF; D6-7: DMEM (Gibco) with 25 mM glucose containing 1:100 B27 (Gibco), 3 nM TTNPB (Sigma); D8: DMEM with 25 mM glucose containing 1:100 B27, 3 nM TTNPB, and 50 ng/ml EGF (R&D Systems); D9-11: DMEM with 25mM glucose containing 1:100 B27 (Stemcell Technologies), 50ng/ml EGF (Peprotech) and 50ng/ml KGF (Peprotech). D12-20: DMEM with 25nM glucose supplemented with 1:100 GlutaMax (Gibco), 1:100 Non-essential Amino Acid (Sigma), 1:100 B27, 10ug/ml Heparin (Sigma), 10uM $ZnSO_4$ (Sigma), 1mM N-Cysteine (Sigma), 10uM ALK5 inhibitor II (Axxora), 1uM γ-secretase inhibitor XX (Millipore), 1uM 3,3',5-Triiodo-L-thyronine sodium salt (Sigma), 500uM Vitamin C (Sigma), and 500nM LDN-193189 (Stemgent). To produce eBCs (Nair et al., 2019), D20 hESC β-like cells were sorted for INS-GFP expression as described below. The sorted cells were collected and distributed into Aggrewell 400 plates (Stemcell Technologies) with 1 million cells per well to re-aggregate into spheres and cultured further for *in vitro* maturation. The spheres were cultured in CMRL (Gibco) supplemented with 1:100 GlutaMax, 1:100 Non-essential Amino Acid, 10% FBS (Gibco), 10ug/ml Heparin, 10uM $ZnSO_4$, 1mM N-Cysteine, 10uM ALK5 inhibitor II, and 1uM 3,3',5-Triiodo-L-thyronine sodium salt (Nair et al., 2019). On day 27, the resulting enriched beta clusters (eBCs) were collected for downstream experiments. Human islets were from the UCSF Islets and Cellular Production Facility.

Mice

NOD.Cg-Prkdcscid Il2rgtm1Wjl/SzJ mice (NSG) were obtained from Jackson Laboratories. Mice

used in this study were maintained according to protocols approved by the University of California, San Francisco
Committee on Laboratory Animal Resource Center. Mouse kidney capsule grafts have been described previously (Russ et al., 2015).

Flow cytometry

For RNA-seq, GFP+ cell counts and reaggregation experiments, hESC β-like day 20 spheres were collected and allowed to settle by gravity. Clusters were washed once with DPBS (Sigma) and dissociated by lightly tapping the tube and gentle pipetted twice after 8 min incubation in Accumax (Millipore). Cell suspension was filtered and sorted on FACSAriaII (BD Bioscience). Dead cells were excluded by DAPI (Invitrogen) staining. For intracellular staining of transcription factors, clusters were dissociated, fixed, permeabilized and stained for various intracellular markers for analysis on LSRFortessa X20 Dual, as described previously(Nair et al., 2019). Data were analyzed with FlowJo software. Cells obtained from day 5 of the differentiation protocol were used as negative controls as they do not express beta cell markers. Anti-human C-peptide antibodies were conjugated in-house using the Molecular Probes Antibody Labeling Kits according to manufacturer's instructions. Antibody details are listed below.

Antibody	Manufacturer	Dilution	Catalog #
Human PAX6-Alexa 647	BD Bioscience	1:50	562249
Islet-1- PE	BD Bioscience	1:50	562547
NKX6.1- Alexa 647	BD Bioscience	1:50	563338
NKX2.2-PE	BD Bioscience	1:50	564730
NeuroD1- Alexa 647	BD Bioscience	1:50	563566
PDX1-PE	BD Bioscience	1:50	562161
C-peptide-488	Chemicon (Mouse antibody) conjugated in house	1:200	C-PEP-01

Cell lines

The INS-GFP MEL-1 line was kindly provided by Ed Stanley (Micallef et al., 2012). The iCrLIN28B line is an INS-GFP MEL-1 line modified with a doxycycline inducible Cas9 and U6 expressed guide RNAs directed toward either end of exon 3 (Fig. 3A). Specifically, a cassette

carrying TRE-Cas9, two U6-gRNAs, and t2A-PURO was targeted to one hAAVS1 allele, while a CAG-M2rtTA, t2A-NEO cassette was targeted to the second hAAVS1 using TALEN driven recombineering as previously described (Gonzalez et al., 2014). The gRNA sequences were: AGAAAATCCGAAGATTTAGG and CTACAGAAAAGAAAACCAAAGGG.. The iLET-7 line is an INS-GFP MEL-1 line modified with doxycycline inducible let-7a/f/b overexpression cassette. The cassette consisted of 3 repeats the pre-let7a/f and pre-let7b hairpins with spacers in between (Fig. 4A). The loop regions of the pre-let-7f and pre-let7b hairpins were replaced with the pre-miR-21 loop to block LIN28A/B recognition and suppression of let-7 biogenesis (Piskounova et al., 2011). The let-7a loop is already resistant to LIN28A/B regulation and thus was left intact (Triboulet et al., 2015). The cassette was placed downstream of a TRE and targeted to AAVS1 locus as described above. Doxycycline was added at a final concentration of 2ug/ml when needed.

Small RNA-seq and RNA-seq

Total RNA was extracted with micro RNeasy kit (Qiagen), treated with DNase I kit (Qiagen) and quantified with Qubit (Invitrogen). Bioanalyzer (Agilent) was employed to control RNA quality. Small RNA-seq libraries were made as previously described (Hafner et al., 2012). RNA-seq libraries were made by using Smart-Seq v4 Ultra Low Input RNA Kit for Sequencing (Takara) and Nextera XT DNA Library Preparation kit (Illumina) thereafter.

Small RNA-seq and RNA-seq data analysis

For RNA-Seq analysis, the data were preprocessed using Kallisto (Bray et al., 2016) and index to Gencode Version 24. For the miRNA-Seq analysis data were preprocessed using CutAdapt v1.8 (DOI:10.14806/ej.17.1.200) to demultiplex and trim adapters, sequences were then aligned using Bowtie v1.1.2 (-n 0 -l 18 –best) to a hairpin genome downloaded from miRbase (Langmead et al., 2009). Differential expression analysis was performed using in R using the Limma-Voom analysis (Liu et al., 2015; Ritchie et al., 2015). Cutoffs for significance were set at an adjusted $p < 0.05$. Plots were generated using the tidyverse and the ggplot2 R package. Let-7 targets were obtained from the TargetScan Release 7.1:June 2016 let-7-5p/98-5p list. GSEA analysis was performed using the current release (16-Jul-2018) from http://www.gsea-msigdb.org/gsea/index.jsp (Mootha et al., 2003; Subramanian et al., 2005).The GseaPreranked tool was used on the log-fold change

ranked miRNAs. The gene sets analyzed were the juvenile vs. adult beta enriched genes from (Arda et al., 2016).

qRT-PCR

Total RNA was extracted with micro RNeasy kit (Qiagen), treated with DNase I kit (Qiagen) and reverse-transcribed using SuperScript III kit (Invitrogen) as per manufacturer's instructions. mRNA qPCR primer sequences are listed below.

Gene	Forward primers	Reverse primers
GAPDH	TGCACCACCAACTGCTTAGC	GGCATGGACTGTGGTCATGAG
LIN28B	AGCCCCTGTTTAGGAAGTGAAA	CACCACAGTTGTAGCATCTATCTCC
CAS9	CCGAAGAGGTCGTGAAGAAG	GCCTTATCCAGTTCGCTCAG
PDX1	AGTGGGCAGGCGGCG	CAACATGACAGCCAGCTCCA
NKX6.1	CACGAGACCCACTTTTTCCG	ACCAGACCTTGACCTGACTCT
NKX2.2	GTCCGGAGGAAGAGAACGAG	CCGTGCAGGGAGTACTGAAG
NERUROD1	ATAGACCTGCTAGCCCCTCA	TGGTCATGTTTCGATTTCCTTTGTT
PAX6	GTCCATCTTTGCTTGGGAAA	TAGCCAGGTTGCGAAGAACT
MAFA	TTGAGCGGAGAACGGTGATT	CGAAGGTGGGAACGGAGAAC
SOX9	CTCTGGAGACTTCTGAACGAGAG	CCTTGAAGATGGCGTTGGGG
ISL1	GGATTTGGAATGGCATGCGG	CATTTGATCCCGTACAACCTGA
HMGA2	ACCCAGGGGAAGACCCAAA	CCTCTTGGCCGTTTTTCTCCA

MiRNA qRT-PCR has been described previously (Moltzahn et al., 2011). The primers and probes for miRNA qRT-PCR are listed below. qRT-PCRs were performed on ABI 7900 system.

miRNA	Stem-loop reverse primers	Forward primers	Dual-labeled Probes
**hsa-let-7a**	CTCAACTGGTGTCGTGGAGTCGGCAATTCAGTTGAGAACTATAC	ACACTCCAGCTGGGTGAGGTAGTAGGTTGT	/56-FAM/TTCAGTTGAGAACTATAC/3IABLFQ/
**hsa-let-7b**	CTCAACTGGTGTCGTGGAGTCGGCAATTCAGTTGAGAACCACAC	ACACTCCAGCTGGGTGAGGTAGTAGGTTGT	/56-FAM/TTCAGTTGAGAACCACAC/3IABLFQ/
**hsa-let-7c**	CTCAACTGGTGTCGTGGAGTCGGCAATTCAGTTGAGAACCATAC	ACACTCCAGCTGGGTGAGGTAGTAGGTTGT	/56-FAM/TTCAGTTGAGAACCATAC/3IABLFQ/
**hsa-let-7d**	CTCAACTGGTGTCGTGGAGTCGGCAATTCAGTTGAGAACTATGC	ACACTCCAGCTGGGAGAGGTAGTAGGTTGC	/56-FAM/TTCAGTTGAGAACTATGC/3IABLFQ/
**hsa-let-7f**	CTCAACTGGTGTCGTGGAGTCGGCAATTCAGTTGAGAACTATAC	ACACTCCAGCTGGGTGAGGTAGTAGATTGT	/56-FAM/TTCAGTTGAGAACTATAC/3IABLFQ/
**hsa-let-7g**	CTCAACTGGTGTCGTGGAGTCGGCAATTCAGTTGAGAACTGTAC	ACACTCCAGCTGGGTGAGGTAGTAGTTTGT	/56-FAM/TTCAGTTGAGAACTGTAC/3IABLFQ/
**Snord15**	CTCAACTGGTGTCGTGGAGTCGGCAATTCAGTTGAGCCTTCTCA	ACACTCCAGCTGGGAGAGGCATTTGTCTGA	/56-FAM/TTCAGTTGAGCCTTCTCA /3IABKFQ/

Western Blots

For total protein extraction, cells/clusters were lysed with RIPA buffer (Thermo Scientific) with protease inhibitors (Roche) on ice. The supernatant was collected for Western blots. Proteins were quantified with BCA protein assay kit (Thermofisher). The proteins were resolved on 4-15% Mini-PROTEAN TGX gels (Bio-rad). Approximately 40ug protein were loaded per lane. The following antibodies were used for blotting: 1:1000 LIN28B (Cell Signaling Technology #4196), 1:5000 GAPDH (Santa Cruz Biotechnology sc-47724). Primary antibody incubation was performed at 4°C overnight. Secondary antibodies (LI-COR) were used at a concentration of 1:10,000. Imaging was performed using an Odyssey LICOR scanner. Quantification was performed using ImageJ software. Antibody details are listed below.

Antibody	Manufacturer	Dilution	Catalog #
LIN28B	Cell Signaling Technology	1:1000	4196
GAPDH	Santa Cruz Biotechnology	1:5000	sc-47724

T7 Endonuclease I assay (T7EI assay)

T7EI assay was employed to assess genome modification. Genomic DNA was extracted with genomic DNA purification kit (Thermofisher) as per manufacturer's instructions. Genomic regions flanking the CRSIPR target sites were PCR amplified with *LIN28B* T7 primers (forward: AAAACTTTAGCTGGACTCTGCAT; reverse: GCTGAAGGCTCAGTTCAGTACAT). PCR products were purified with PCR purification kit (Qiagen). For T7EI assays, 200ng of purified PCR products were denatured and reannealed in NEB buffer 2 (New England Biolabs) in a total volume of 19ul using the following protocol: 95°C, 5min; 95°C-85°C at -2°C/s; 85°C-25°C at -0.1°C/s; hold at 12°C. The hybridized PCR products were then treated with 1ul of T7EI (New England Biolabs) at 37°C for 20min in 20ul final reaction volume. Products were then 1:10 diluted and analyzed with High Sensitivity D1000 ScreenTape System (Agilent) on TapeStation 2200 (Agilent) according to manufacturer's instructions. Quantification was based on TapeStation readout of peak molarity. Indel percentage was determined by the formula: %gene modification=100*(1-(1-fraction cleaved)$^{1/2}$)

Glucose stimulation insulin secretion assays

For static insulin secretion assays, hESC eBCs were equally distributed into 8-strip tubes and washed twice with Krebs-Ringer Bicarbonate buffer (KRB) containing 2.8mM glucose. Samples were incubated for half an hour in KRB containing 2.8mM glucose to allow equilibration of cells. The buffer was removed and replaced with fresh KRB containing 2.8nM glucose for 30min incubation followed by 30min incubation in KRB containing 16.7mM glucose and then another 30min incubation in KRB with 30mM KCl. After each incubation period, supernatant was collected for human C-peptide-specific ELISA with C-peptide Chemiluminesence ELISA kit (ALPCO) as per manufacturer's instructions.

For dynamic insulin secretion assays, eBCs were assayed using the perifusion system from Biorep technologies. The clusters were placed on filters in plastic chambers that were maintained at 37°C in a temperature controlled environment. Under temperature- and CO_2-controlled conditions, the clusters were perifused at 100 ul min^{-1} with Krebs-Ringer buffer (KRB). After an initial 1.5 hour long preincubation in 2.8mM glucose-KRB, alternating low (2.8mM) and high (20mM) glucose were perfused through the system. Flow-through was collected over the course of the experiment, and C-peptide levels were measured using the STELLUX® Chemi Human C-peptide ELISA kit (Alpco). For the static assays, spheres were equally distributed into 8-strip tubes and washed twice with 2.8mM glucose-KRB. Samples were incubated for half an hour in KRB containing 2.8mM glucose to allow equilibration of cells. The buffer was removed and replaced with fresh KRB containing 2.8mM glucose for 30min incubation followed by 30min incubation in KRB containing 16.7mM glucose. After each incubation period, supernatant was collected for human C-peptide-specific ELISA with the STELLUX® Chemi Human C-peptide ELISA kit (Alpco) as per manufacturer's instructions.

Statistics

Statistical tests performed for specific data sets are described in the corresponding figure legends. In brief, under the assumption of normal distribution, two-tailed unpaired *t*-tests (Student's *t*-test) were used if standard deviation (SD) was equal or two-tailed unpaired *t*-tests with Welch's correction were used if SD was unequal for pairwise comparison in Figures1D, 2G, 3C~F, 4C~F, S2C~E, S3, S4E; two-tailed paired *t*-tests were used in Figure 3B. All statistical tests were

performed in GraphPad Prism Software v7. Statistical significance of the sequencing data was calculated by the Limma package using linear modeling and empirical Bayes statistics implemented in the lmFit and eBayes functions. Statistical methods were not used to determine sample size.

Accession number: The GEO accession number for the genomic data presented is GSE108654.

Supplemental References:

Arda, H.E., Li, L., Tsai, J., Torre, E.A., Rosli, Y., Peiris, H., Spitale, R.C., Dai, C., Gu, X., Qu, K., *et al.* (2016). Age-Dependent Pancreatic Gene Regulation Reveals Mechanisms Governing Human beta Cell Function. Cell Metab *23*, 909-920.

Bray, N.L., Pimentel, H., Melsted, P., and Pachter, L. (2016). Near-optimal probabilistic RNA-seq quantification. Nat Biotechnol *34*, 525-527.

Czechanski, A., Byers, C., Greenstein, I., Schrode, N., Donahue, L.R., Hadjantonakis, A.K., and Reinholdt, L.G. (2014). Derivation and characterization of mouse embryonic stem cells from permissive and nonpermissive strains. Nat Protoc *9*, 559-574.

Gonzalez, F., Zhu, Z., Shi, Z.D., Lelli, K., Verma, N., Li, Q.V., and Huangfu, D. (2014). An iCRISPR platform for rapid, multiplexable, and inducible genome editing in human pluripotent stem cells. Cell Stem Cell *15*, 215-226.

Hafner, M., Renwick, N., Farazi, T.A., Mihailovic, A., Pena, J.T., and Tuschl, T. (2012). Barcoded cDNA library preparation for small RNA profiling by next-generation sequencing. Methods *58*, 164-170.

Langmead, B., Trapnell, C., Pop, M., and Salzberg, S.L. (2009). Ultrafast and memory-efficient alignment of short DNA sequences to the human genome. Genome Biol *10*, R25.

Liu, R., Holik, A.Z., Su, S., Jansz, N., Chen, K., Leong, H.S., Blewitt, M.E., Asselin-Labat, M.L., Smyth, G.K., and Ritchie, M.E. (2015). Why weight? Modelling sample and observational level variability improves power in RNA-seq analyses. Nucleic Acids Res *43*, e97.

Micallef, S.J., Li, X., Schiesser, J.V., Hirst, C.E., Yu, Q.C., Lim, S.M., Nostro, M.C., Elliott, D.A., Sarangi, F., Harrison, L.C., *et al.* (2012). INS(GFP/w) human embryonic stem cells facilitate isolation of in vitro derived insulin-producing cells. Diabetologia *55*, 694-706.

Moltzahn, F., Hunkapiller, N., Mir, A.A., Imbar, T., and Blelloch, R. (2011). High throughput microRNA profiling: optimized multiplex qRT-PCR at nanoliter scale on the fluidigm dynamic arrayTM IFCs. Journal of visualized experiments : JoVE.

Mootha, V.K., Lindgren, C.M., Eriksson, K.F., Subramanian, A., Sihag, S., Lehar, J., Puigserver, P., Carlsson, E., Ridderstrale, M., Laurila, E.*, et al.* (2003). PGC-1alpha-responsive genes involved in oxidative phosphorylation are coordinately downregulated in human diabetes. Nat Genet *34*, 267-273.

Nair, G.G., Liu, J.S., Russ, H.A., Tran, S., Saxton, M.S., Chen, R., Juang, C., Li, M.-l., Nguyen, V.Q., Giacometti, S.*, et al.* (2019). Recapitulating endocrine cell clustering in culture promotes maturation of human stem-cell-derived β cells. Nature cell biology *21*, 263-274.

Piskounova, E., Polytarchou, C., Thornton, J.E., LaPierre, R.J., Pothoulakis, C., Hagan, J.P., Iliopoulos, D., and Gregory, R.I. (2011). Lin28A and Lin28B inhibit let-7 microRNA biogenesis by distinct mechanisms. Cell *147*, 1066-1079.

Ritchie, M.E., Phipson, B., Wu, D., Hu, Y., Law, C.W., Shi, W., and Smyth, G.K. (2015). limma powers differential expression analyses for RNA-sequencing and microarray studies. Nucleic Acids Res *43*, e47.

Russ, H.A., Parent, A.V., Ringler, J.J., Hennings, T.G., Nair, G.G., Shveygert, M., Guo, T., Puri, S., Haataja, L., Cirulli, V.*, et al.* (2015). Controlled induction of human pancreatic progenitors produces functional beta-like cells in vitro. EMBO J *34*, 1759-1772.

Subramanian, A., Tamayo, P., Mootha, V.K., Mukherjee, S., Ebert, B.L., Gillette, M.A., Paulovich, A., Pomeroy, S.L., Golub, T.R., Lander, E.S.*, et al.* (2005). Gene set enrichment analysis: a knowledge-based approach for interpreting genome-wide expression profiles. Proc Natl Acad Sci U S A *102*, 15545-15550.

Triboulet, R., Pirouz, M., and Gregory, R.I. (2015). A Single Let-7 MicroRNA Bypasses LIN28-Mediated Repression. Cell Rep *13*, 260-266.
